# Dissociative and prioritized modeling of behaviorally relevant neural dynamics using recurrent neural networks

**DOI:** 10.1038/s41593-024-01731-2

**Published:** 2024-09-06

**Authors:** Omid G. Sani, Bijan Pesaran, Maryam M. Shanechi

**Affiliations:** 1 Ming Hsieh Department of Electrical and Computer Engineering, Viterbi School of Engineering, University of Southern California, Los Angeles, CA USA; 2 Perelman School of Medicine, University of Pennsylvania, Philadelphia, PA USA; 3 Thomas Lord Department of Computer Science, University of Southern California, Los Angeles, CA USA; 4 Neuroscience Graduate Program, University of Southern California, Los Angeles, CA USA; 5 Alfred E. Mann Department of Biomedical Engineering, University of Southern California, Los Angeles, CA USA

**Keywords:** Dynamical systems, Machine learning, Neural encoding, Neural decoding, Brain-machine interface

## Abstract

Understanding the dynamical transformation of neural activity to behavior requires new capabilities to nonlinearly model, dissociate and prioritize behaviorally relevant neural dynamics and test hypotheses about the origin of nonlinearity. We present dissociative prioritized analysis of dynamics (DPAD), a nonlinear dynamical modeling approach that enables these capabilities with a multisection neural network architecture and training approach. Analyzing cortical spiking and local field potential activity across four movement tasks, we demonstrate five use-cases. DPAD enabled more accurate neural–behavioral prediction. It identified nonlinear dynamical transformations of local field potentials that were more behavior predictive than traditional power features. Further, DPAD achieved behavior-predictive nonlinear neural dimensionality reduction. It enabled hypothesis testing regarding nonlinearities in neural–behavioral transformation, revealing that, in our datasets, nonlinearities could largely be isolated to the mapping from latent cortical dynamics to behavior. Finally, DPAD extended across continuous, intermittently sampled and categorical behaviors. DPAD provides a powerful tool for nonlinear dynamical modeling and investigation of neural–behavioral data.

## Abstract (web summary)

The authors present DPAD, a deep learning method, for dynamical neural–behavioral modeling. It dissociates behaviorally relevant neural dynamics, better predicts neural–behavioral data and reveals insight into where their nonlinearities can be isolated.

## Main

Understanding how neural population dynamics give rise to behavior is a major goal in neuroscience. Many methods that relate neural activity to behavior use static mappings or embeddings, which do not describe the temporal structure in how neural population activity evolves over time^1^. In comparison, dynamical models can describe these temporal structures in terms of low-dimensional latent states embedded in the high-dimensional space of neural recordings. Prior dynamical models have often been linear or generalized linear^1–7^, thus motivating recent work to develop support for piece-wise linear^8^, locally linear^9^, switching linear^10–13^ or nonlinear^14–27^ models of neural dynamics, especially in applications such as single-trial smoothing of neural population activity^9,14–19^ and decoding behavior^20–24,26^. Once trained, the latent states of these models can subsequently be mapped to behavior^1,25^ to learn an overall dynamical transformation from neural activity to behavior. However, multiple challenges hinder the dynamical modeling and interpretation of neural–behavioral transformations.

First, the neural–behavioral transformation can exhibit nonlinearities, which the dynamical model should capture. Moreover, these nonlinearities can be in one or more different elements within the dynamical model, for example, in the dynamics of the latent state or in its embedding. Enabling hypothesis testing regarding the origin of nonlinearity (that is, where the nonlinearity can be isolated to within the model) is important for interpreting neural computations and developing neurotechnology but remains largely unaddressed in current nonlinear models. Second, neural dynamics related to a given behavior often constitute a minority of the total neural variance^28–33^. To avoid missing or confounding these dynamics, nonlinear dynamical models need to dissociate behaviorally relevant neural dynamics from other neural dynamics and prioritize the learning of the former, which is currently not possible. Indeed, existing nonlinear methods for modeling neural activity either do not explicitly model temporal dynamics^34–36^ or do not prioritize behaviorally relevant dynamics^16,37,38^, or have a mixed objective^18^ that may mix behaviorally relevant and other neural dynamics in the same latent states (Discussion and Extended Data Table 1). Our prior method, termed PSID^6^, has enabled prioritized dissociation of behaviorally relevant neural dynamics but for linear dynamical models. Third, for broad applicability, in addition to continuous behaviors, dynamical models should admit categorical (for example, choices) or intermittently sampled behaviors (for example, mood reports), which are not supported by existing dynamical methods with a mixed objective^18^ or by PSID. To date, learning nonlinear dynamical models of neural population activity that can address the above challenges has not been achieved.

**Fig. 1:**
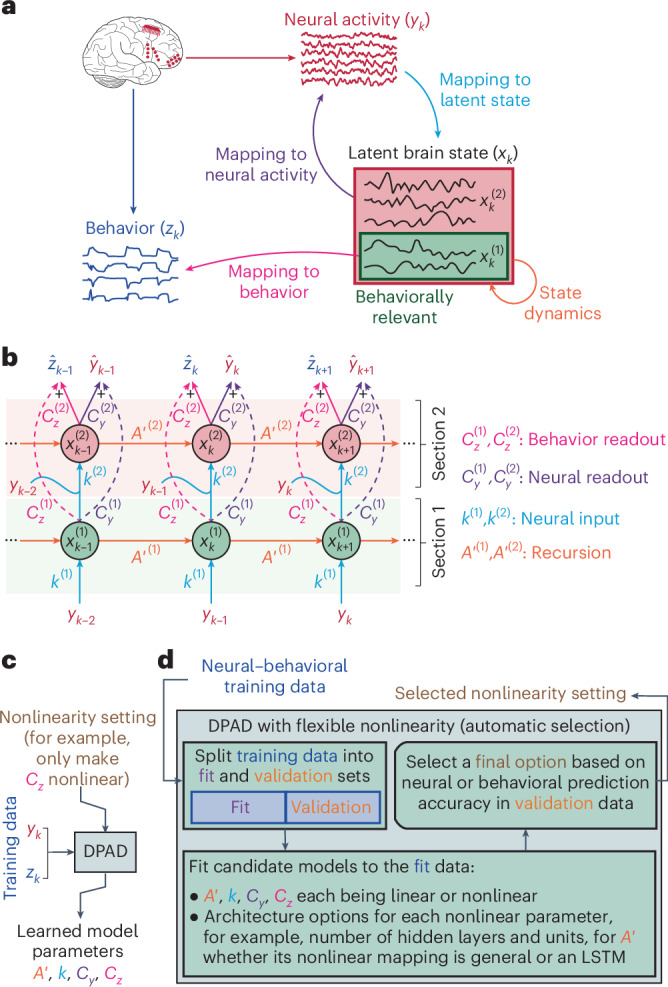
DPAD overview. **a**, DPAD decomposes the neural–behavioral transformation into four interpretable mapping elements. It learns the mapping of neural activity (*y*_*k*_) to latent states (*x*_*k*_), termed neural input in the model; learns the dynamics or temporal structure of the latent states, termed recursion in the model; dissociates the behaviorally relevant latent states (${x}_{k}^{\left(1\right)}$) that are relevant to a measured behavior (*z*_*k*_) from other states (${x}_{k}^{\left(2\right)}$); learns the mapping of the latent states to behavior and to neural activity, termed behavior and neural readouts in the model; and allows flexible linear or nonlinear mappings in any of its elements. DPAD additionally prioritizes the learning of behaviorally relevant neural dynamics to learn them accurately. **b**, Computation graph of the DPAD model consists of a two-section RNN whose input is neural activity at the current time step and whose outputs are the predicted behavior and neural activity in the next time step (Methods). This graph assumes that computations are Markovian, that is, with a high enough dimension, latent states can summarize the information from past neural data that is useful for predicting future neural–behavioral data. Each of the four mapping elements from **a** has a corresponding parameter in each section of the RNN model, indicated by the same colors and termed as introduced in **a**. **c**, We developed a four-step optimization method to learn all the model parameters from training neural–behavioral data (Supplementary Fig. 1a). Further, each model parameter can be specified via the ‘nonlinearity setting’ to be linear or nonlinear with various options to implement the nonlinearity (Supplementary Fig. 1b,c). After a model is learned, only past neural activity is used to decode behavior and predict neural activity using the computation graph in **b**. **d**, DPAD also has the option of automatically selecting the ‘nonlinearity setting’ for the data by fitting candidate models and comparing them in terms of both behavior decoding and neural self-prediction accuracy (Methods). In this work, we chose among 90 candidate models with various nonlinearity settings (Methods). We refer to this automatic selection of nonlinearity as ‘DPAD with flexible nonlinearity’.

Here, we develop dissociative prioritized analysis of dynamics (DPAD), a nonlinear dynamical modeling framework using recurrent neural networks (RNNs) that addresses all the above challenges. DPAD models both behaviorally relevant and other neural dynamics but dissociates them into separate latent states and prioritizes the learning of the former. To do so, we formulate a two-section RNN as the DPAD nonlinear dynamical model and develop a four-step optimization algorithm to train it. The first RNN section learns the behaviorally relevant latent states with priority, and the second section learns any remaining neural dynamics (Fig. 1a and Supplementary Fig. 1). Moreover, DPAD adjusts these optimization steps as needed to admit continuous-valued, categorical or intermittently sampled data (Methods). Furthermore, to capture nonlinearity in the neural–behavioral transformation and enable hypothesis testing regarding its origins, DPAD decomposes this transformation into the following four interpretable elements and allows each element to become linear or nonlinear (Fig. 1a,b): the mapping from neural activity to the latent space (neural input), the latent state dynamics within this space (recursion) and the mappings of the state to neural activity and behavior (neural and behavior readouts). Finally, we formulate the DPAD model in predictor form such that the learned model can be directly used for inference, enabling causal and computationally efficient decoding for data, whether with or without a fixed-length trial structure (Methods).

To show its broad utility, we demonstrate five distinct use-cases for DPAD across four diverse nonhuman primate (NHP) datasets consisting of both population spiking activity and local field potentials (LFPs). First, DPAD more accurately models the overall neural–behavioral data than alternative nonlinear and linear methods. This is due both to DPAD’s prioritized and dynamical modeling of behaviorally relevant neural dynamics and to its nonlinearity. Second, DPAD can automatically uncover nonlinear dynamical transformations of raw LFP that are more predictive of behavior than traditional LFP power band features and in some datasets can even outperform population spiking activity in terms of behavior prediction. Further, DPAD reveals that among the neural modalities, the degree of nonlinearity is greatest for the raw LFP. Third, DPAD enables nonlinear and dynamical neural dimensionality reduction while preserving behavior information, thus extracting lower-dimensional yet more behavior-predictive latent states from past neural activity. Fourth, DPAD enables hypothesis testing regarding the origin of nonlinearity in the neural–behavioral transformation. Consistently across our movement-related datasets, doing so revealed that summarizing the nonlinearities just in the behavior readout from the latent state is largely sufficient for predicting the neural–behavioral data (see Discussion). Fifth, DPAD extends to categorical and intermittently observed behaviors, which is important for cognitive neuroscience^11,39^ and neuropsychiatry^40–42^. Together, these results highlight DPAD’s broad utility as a dynamical modeling tool to investigate the nonlinear and dynamical transformation of neural activity to specific behaviors across various domains of neuroscience.

## Results

### Overview of DPAD

#### Formulation

We model neural activity and behavior jointly and nonlinearly (Methods) as

1 $$\left\{\begin{array}{c}{x}_{k+1}={A}^{{\prime} }\left({x}_{k}\right)+K\left({y}_{k}\right)\\ {y}_{k}={C}_{y}\left({x}_{k}\right)+{e}_{k}\\ {z}_{k}={C}_{z}\left({x}_{k}\right)+{\epsilon }_{k}\end{array},\right.$$

where *k* is the time index, ${y}_{k}\in {{\mathbb{R}}}^{{n}_{y}}$ and ${z}_{k}\in {{\mathbb{R}}}^{{n}_{z}}$ denote the neural activity and behavior time series, respectively, ${x}_{k}\in {{\mathbb{R}}}^{{n}_{x}}$ is the latent state, and *e*_*k*_ and ${{\epsilon }}_{k}$ denote neural and behavior dynamics that are unpredictable from past neural activity. Multi-input–multi-output functions *A*′ (recursion), *K* (neural input), *C*_*y*_ (neural readout) and *C*_*z*_ (behavior readout) are parameters that fully specify the model and have interpretable descriptions (Methods, Supplementary Note 1 and Fig. 1a,b). The adjusted formulation for intermittently sampled and noncontinuous-valued (for example, categorical) data is provided in Methods. DPAD supports both linear and nonlinear modeling, which will be termed linear DPAD and nonlinear DPAD (or just DPAD), respectively.

#### Dissociative and prioritized learning

We further expand the model in Eq. (1) in two sections, as depicted in Fig. 1b (Eq. (2) in Methods and Supplementary Note 2). The first and second sections describe the behaviorally relevant neural dynamics and the other neural dynamics with latent states ${x}_{k}^{(1)}\in {{\mathbb{R}}}^{{n}_{1}}$ and ${x}_{k}^{(2)}\in {{\mathbb{R}}}^{{n}_{x}-{n}_{1}}$, respectively. We specify the parameters of the two RNN sections with superscripts (for example, *K*^(1)^ and *K*^(2)^) and learn them all sequentially via a four-step optimization (Methods, Supplementary Fig. 1a and Fig. 1b). The first two steps exclusively learn neural dynamics that are behaviorally relevant with the objective of behavior prediction, whereas the optional last two steps learn any remaining neural dynamics with the objective of residual neural prediction (Methods and Supplementary Fig. 1). We implement DPAD in Tensorflow and use an ADAM^43^ optimizer (Methods).

#### Comparison baselines

As a baseline, we compare DPAD with standard nonlinear RNNs fitted to maximize neural prediction, unsupervised with respect to behavior. We refer to this baseline as nonlinear neural dynamical modeling (NDM)^6^ or as linear NDM if all RNN parameters are linear. NDM is nondissociative and nonprioritized, so comparisons with NDM show the benefit of DPAD’s prioritized dissociation of behaviorally relevant neural dynamics. We also compare DPAD with latent factor analysis via dynamical systems (LFADS)^16^ and with two concurrently^44^ developed methods with DPAD named targeted neural dynamical modeling (TNDM)^18^ and consistent embeddings of high-dimensional recordings using auxiliary variables (CEBRA)^36^ in terms of neural–behavioral prediction; however, as summarized in Extended Data Table 1, these and other existing methods differ from DPAD in key goals and capabilities and do not enable some of DPAD’s use-cases (see Discussion).

#### Decoding using past neural data

Given DPAD’s learned parameters, the latent states can be causally extracted from neural activity by iterating through the RNN in Eq. (1) (Methods and Supplementary Note 1). Note that this decoding always only uses neural activity without seeing the behavior data.

#### Flexible control of nonlinearities

We allow each model parameter (for example, *C*_*z*_) to be an arbitrary multilayer neural network (Supplementary Fig. 1c), which can universally approximate any smooth nonlinear function or implement linear matrix multiplications (Methods and Supplementary Fig. 1b). Users can manually specify which parameters will be learned as nonlinear and with what architecture (Fig. 1c; see application in use-case 4). Alternatively, DPAD can automatically determine the best nonlinearity setting for the data by conducting a search over nonlinearity options (Fig. 1d and Methods), a process that we refer to as flexible nonlinearity. For a fair comparison, we also implement this flexible nonlinearity for NDM. To show the benefits of nonlinearity, we also compare with linear DPAD, where all parameters are set to be linear, in which case Eq. (1) formulates a standard linear state-space model in predictor form (Methods).

#### Evaluation metrics

We evaluate how well the models can use the past neural activity to predict the next sample of behavior (termed ‘decoding’) or the next sample of neural activity itself (termed ‘neural self-prediction’ or simply ‘self-prediction’). Thus, decoding and self-prediction assess the one-step-ahead prediction accuracies and reflect the learning of behaviorally relevant and overall neural dynamics, respectively. Both performance measures are always computed with cross-validation (Methods).

Our primary interest is to find models that simultaneously reach both accurate behavior decoding and accurate neural self-prediction. But in some applications, only one of these metrics may be of interest. Thus, we use the term ‘performance frontier’ to refer to the range of performances achievable by those models that compared to every other model are better in neural self-prediction and/or behavior decoding or are similar in terms of both metrics (Methods).

### Diverse neural–behavioral datasets

**Fig. 2:**
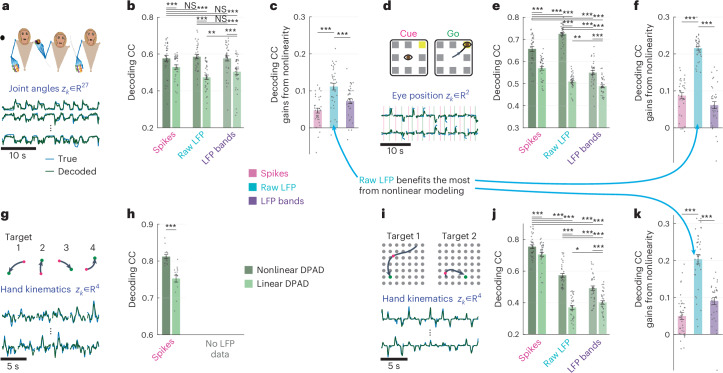
DPAD learns more accurate models of behaviorally relevant neural dynamics for all neural modalities by capturing nonlinearities, with raw LFP activity benefiting the most from nonlinear modeling. **a**, The 3D reach task, along with example true and decoded behavior dimensions, decoded from spiking activity using DPAD, with more example trajectories for all modalities shown in Supplementary Fig. 3. **b**, Cross-validated decoding accuracy correlation coefficient (CC) achieved by linear and nonlinear DPAD. Results are shown for spiking activity, raw LFP activity and LFP band power activity (Methods). For nonlinear DPAD, the nonlinearities are selected automatically based on the training data to maximize behavior decoding accuracy (that is, flexible nonlinearity). The latent state dimension in each session and fold is chosen (among powers of 2 up to 128) as the smallest that reaches peak decoding in the training data among all state dimensions (Methods). Bars show the mean, whiskers show the s.e.m., and dots show all data points (*N* = 35 session-folds). Asterisks (*) show significance level for a one-sided Wilcoxon signed-rank test (*P < 0.05, **P < 0.005 and ***P < 0.0005); NS, not significant. **c**, The difference between the nonlinear and linear results from **b** shown with the same notations. **d**–**f**, Same as **a**–**c** for the second dataset with saccadic eye movements (*N* = 35 session-folds). **g**,**h**, Same as **a** and **b** for the third dataset, which did not include LFP data, with sequential cursor reaches controlled via a 2D manipulandum (*N* = 15 session-folds). Behavior consists of the 2D position and velocity of the cursor, denoted as ‘hand kinematics’ in the figure. **i**–**k**, Same as **a**–**c** for the fourth dataset, with random grid virtual reality cursor reaches controlled via fingertip movement (*N* = 35 session-folds). For all DPAD variations, only the first two optimization steps were used in this figure (that is, *n*_1_ = *n*_*x*_) to only focus on learning behaviorally relevant neural dynamics. Source data

We used DPAD to study the behaviorally relevant neural dynamics in four NHPs performing four different tasks (Fig. 2 and Methods). In the first task, the animal made naturalistic three-dimensional (3D) reach, grasp and return movements to diverse locations while the joint angles in the arm, elbow, wrist and fingers were tracked as the behavior (Fig. 2a)^6,45^. In the second task, the animal made saccadic eye movements to one of eight possible targets on a screen, with the two-dimensional (2D) eye position tracked as the behavior (Fig. 2d)^6,46^. In the third task, the animal made sequential 2D reaches on a screen using a cursor controlled with a manipulandum while the 2D cursor position and velocity were tracked as the behavior (Fig. 2g)^47,48^. In the fourth task, the animal made 2D reaches to random targets in a virtual-reality-presented grid via a cursor that mirrored the animal’s fingertip movements, for which the 2D position and velocity were tracked as the behavior (Fig. 2i)^49^. In tasks 1 and 4, primary motor cortical activity was modeled. For tasks 2 and 3, prefrontal cortex and dorsal premotor cortical activities were modeled, respectively.

In all datasets, we modeled the Gaussian smoothed spike counts as the main neural modality (Methods). In three datasets that had LFP, we also modeled the following two additional modalities: (1) raw LFP, downsampled to the sampling rate of behavior (that is, 50-ms time steps), which in the motor cortex is known as the local motor potential^50–52^ and has been used to decode behavior^6,50–53^; and (2) LFP power in standard frequency bands from delta (0.1–4 Hz) to high gamma (130–170 Hz (refs. ^5,6,40^); Methods). Similar results held for all three modalities.

### Numerical simulations validate DPAD

**Extended Data Fig. 1:**
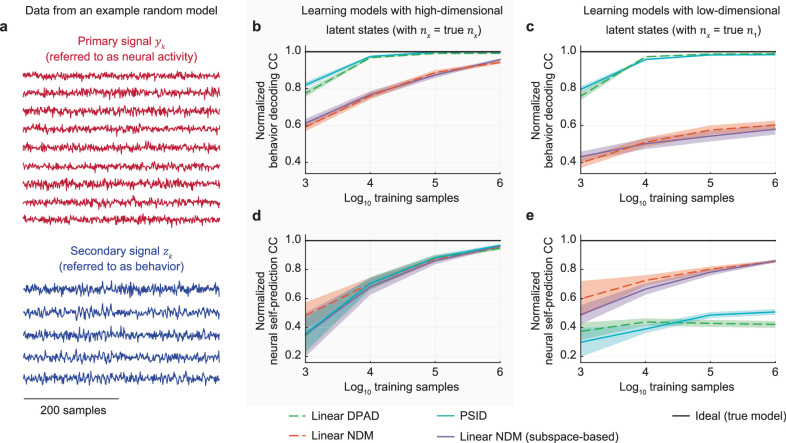
DPAD dissociates and prioritizes the behaviorally relevant neural dynamics while also learning the other neural dynamics in numerical simulations of linear models. **a**, Example data generated from one of 100 random models (Methods). These random models do not emulate real data but for terminological consistency, we still refer to the primary signal (that is, *y*_*k*_ in Eq. (1)) as the ‘neural activity’ and to the secondary signal (that is, *z*_*k*_ in Eq. (1)) as the ‘behavior’. **b**, Cross-validated behavior decoding accuracy (correlation coefficient, CC) for each method as a function of the number of training samples when we use a state dimension equal to the total state dimension of the true model. The performance measures for each random model are normalized by their ideal values that were achieved by the true model itself. Performance for the true model is shown in black. Solid lines and shaded areas are defined as in Fig. 5b (*N* = 100 random models). **c**, Same as **b** but when learned models have low-dimensional latent states with enough dimensions just for the behaviorally relevant latent states (that is, *n*_*x*_ = *n*_1_). **d**-**e**, Same as **b**-**c** showing the cross-validated normalized neural self-prediction accuracy. Linear NDM, which learns the parameters using a numerical optimization, performs similarly to a linear algebraic subspace-based implementation of linear NDM^67^, thus validating NDM’s numerical optimization implementation. Linear DPAD, just like PSID^6^, achieves almost ideal behavior decoding even with low-dimensional latent states (**c**); this shows that DPAD correctly dissociates and prioritizes behaviorally relevant dynamics, as opposed to aiming to simply explain the most neural variance as non-prioritized methods such as NDM do. For this reason, with a low-dimensional state, non-prioritized NDM methods can explain neural activity well (**e**) but prioritized methods can explain behavior much better (**c**). Nevertheless, using the second stage of PSID and the last two optimization steps in DPAD, these two prioritized techniques are still able to learn the overall neural dynamics accurately if state dimension is high enough (**d**). Overall, the performance of linear DPAD and PSID^6^ are similar for the special case of linear modeling.

**Extended Data Fig. 2:**
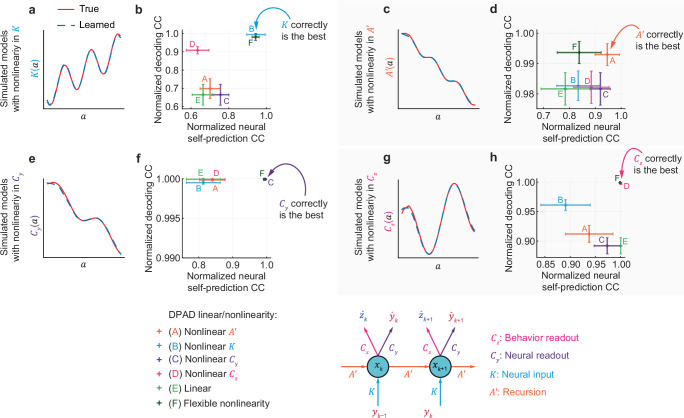
DPAD successfully identifies the origin of nonlinearity and learns it in numerical simulations. DPAD can perform hypothesis testing regarding the origin of nonlinearity by considering both behavior decoding (vertical axis) and neural self-prediction (horizontal axis). **a**, True value for nonlinear neural input parameter *K* in an example random model with nonlinearity only in *K* and the nonlinear value that DPAD learned for this parameter when only *K* in the learned model was set to be nonlinear. The true and learned mappings match and almost exactly overlap. **b**, Behavior decoding and neural self-prediction accuracy achieved by DPAD models with different locations of nonlinearities. These accuracies are for data generated from 20 random models that only had nonlinearity in the neural input parameter *K*. Performance measures for each random model are normalized by their ideal values that were achieved by the true model itself. Pluses and whiskers are defined as in Fig. 3 (*N* = 20 random models). **c**,**d**, Same as **a**,**b** for data simulated from models that only have nonlinearity in the recursion parameter *A*′. **e**-**f**, Same as **a**,**b** for data simulated from models that only have nonlinearity in the neural readout parameter *C*_*y*_. **g**,**h**, Same as **a**,**b** for data simulated from models that only have nonlinearity in the behavior readout parameter *C*_*z*_. In each case (**b**,**d**,**f**,**h**), the nonlinearity option that reaches closest to the upper-rightmost corner of the plot, that is, has both the best behavior decoding and the best neural self-prediction, is chosen as the model that specifies the origin of nonlinearity. Regardless of the true location of nonlinearity (**b**,**d**,**f**,**h**), always the correct location (for example, *K* in **b**) achieves the best performance overall compared with all other locations of nonlinearities. These results provide evidence that by fitting and comparing DPAD models with different nonlinearities, we can correctly find the origin of nonlinearity in simulated data.

We first validate DPAD with linear simulations here (Extended Data Fig. 1) and then present nonlinear simulations under use-case 4 below (Extended Data Fig. 2 and Supplementary Fig. 2). We simulated general random linear models (not emulating any real data) in which only a subset of state dimensions contributed to generating behavior and thus were behaviorally relevant (Methods). We found that with a state dimension equal to that of the true model, DPAD achieved ideal cross-validated prediction (that is, similar to the true model) for both behavior and neural signals (Extended Data Fig. 1b,d). Moreover, even given a minimal state dimension equal to the true behaviorally relevant state dimension, DPAD still achieved ideal prediction for behavior (Extended Data Fig. 1c). Finally, across various regimens of training samples, linear DPAD performed similarly to the linear-algebraic-based PSID^6^ from our prior work (Extended Data Fig. 1). Thus, hereafter, we use linear DPAD as our linear modeling benchmark.

### Use-case 1: DPAD enables nonlinear neural–behavioral modeling across modalities

#### DPAD captures nonlinearity in behaviorally relevant dynamics

We modeled each neural modality (spiking, raw LFP or LFP power) along with behavior using linear and nonlinear DPAD and compared their cross-validated behavior decoding (Fig. 2b,e,h,j and Supplementary Fig. 3). Across all neural modalities in all datasets, nonlinear DPAD achieved significantly higher decoding accuracy than linear DPAD. This result suggests that there is nonlinearity in the dynamical neural–behavioral transformation, which DPAD successfully captures (Fig. 2b,e,h,j).

#### DPAD better predicts the neural–behavioral data

**Fig. 3:**
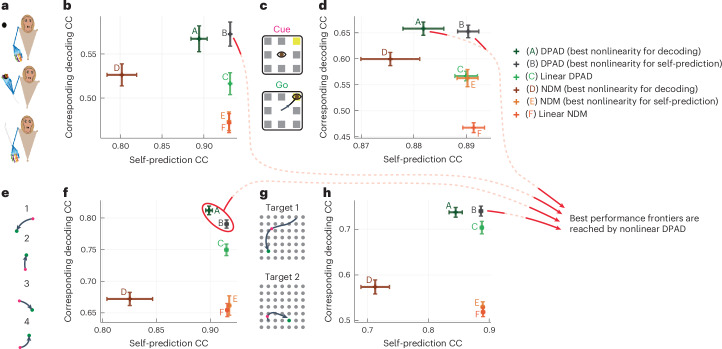
DPAD more accurately learns behaviorally relevant neural dynamics while also capturing overall neural dynamics as accurately as other methods. **a**, The 3D reach task. **b**, Cross-validated neural self-prediction accuracy (CC) achieved by each method shown on the horizontal axis versus the corresponding behavior decoding accuracy on the vertical axis for modeling spiking activity. Latent state dimension for each method in each session, and fold is chosen (among powers of 2 up to 128) as the smallest that reaches peak neural self-prediction in training data or reaches peak decoding in training data, whichever is larger (Methods). The plus on the plot shows the mean self-prediction and decoding accuracy across sessions and folds (*N* = 35 session-folds), and the horizontal and vertical whiskers show the s.e.m. for these two measures, respectively. Capital letter annotations denote the methods according to the legend to make the plots more accessible. Models whose self-prediction and decoding accuracy measures lead to values toward the top-rightmost corner of the plot lie on the best performance frontier (indicated by red arrows) as they have better performance in both measures and thus better explain the neural–behavioral data (Methods). **c**,**d**, Same as **a** and **b** for the second dataset with saccadic eye movements (*N* = 35 session-folds). **e**,**f**, Same as **a** and **b** for the third dataset, with sequential cursor reaches controlled via a 2D manipulandum (*N* = 15 session-folds). **g**,**h**, Same as **a** and **b** for the fourth dataset with random grid virtual reality cursor reaches controlled via fingertip position (*N* = 35 session-folds). For all DPAD variations, the first 16 latent state dimensions are learned using the first two optimization steps, and the remaining dimensions are learned using the last two optimization steps (that is, *n*_1_ = 16). For nonlinear DPAD/NDM, we fit models with different combinations of nonlinearities and then select a final model among these fitted models based on either decoding or self-prediction accuracy in the training data and report both sets of results (Supplementary Fig. 1 and Methods). DPAD with nonlinearity selected based on neural self-prediction was better than all other methods overall (**b**, **d**, **f** and **h**). Source data

**Fig. 4:**
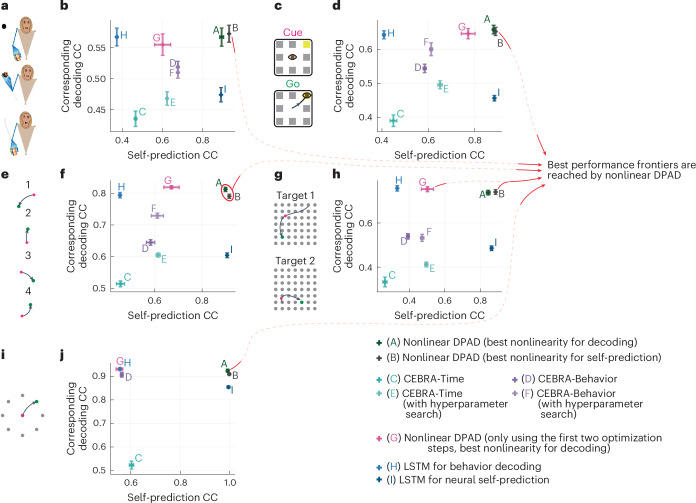
DPAD outperforms various existing methods in neural–behavioral prediction. **a**–**h**, Figure content is parallel to Fig. 3 (with pluses and whiskers defined in the same way) but instead of NDM shows CEBRA and LSTM networks as baselines (Methods). **i**,**j**, Here, we also add a fifth dataset^54^ (Methods), where in each trial an NHP moves a cursor from a center point to one of eight peripheral targets (**i**). In this fifth dataset (*N* = 5 folds), we use the exact CEBRA hyperparameters that were used for this dataset from the paper introducing CEBRA^36^. In the other four datasets (*N* = 35 session-folds in **b**,**d** and **h** and *N* = 15 session-folds in **f**), we also show CEBRA results for when hyperparameters are picked based on an extensive search (Methods). Two types of LSTM networks are shown, one fitted to decode behavior from neural activity and another fitted to predict the next time step of neural activity (self-prediction). We also show the results for DPAD when only using the first two optimization steps. Note that CEBRA-Behavior (denoted by D and F), LSTM for behavior decoding (denoted by H) and DPAD when only using the first two optimization steps (denoted by G) dedicate all their latent states to behavior-related objectives (for example, prediction or contrastive loss), whereas other methods dedicate some or all latent states to neural self-prediction. As in Fig. 3, the final latent dimension for each method in each session and fold is chosen (among powers of 2 up to 128) as the smallest that reaches peak neural self-prediction in training data or reaches peak decoding in training data, whichever is larger (Methods). Across all datasets, DPAD outperforms baseline methods in terms of cross-validated neural–behavioral prediction and lies on the best performance frontier. For a summary of the fundamental differences in goals and capabilities of these methods, see Extended Data Table 1. Source data

**Extended Data Fig. 3:**
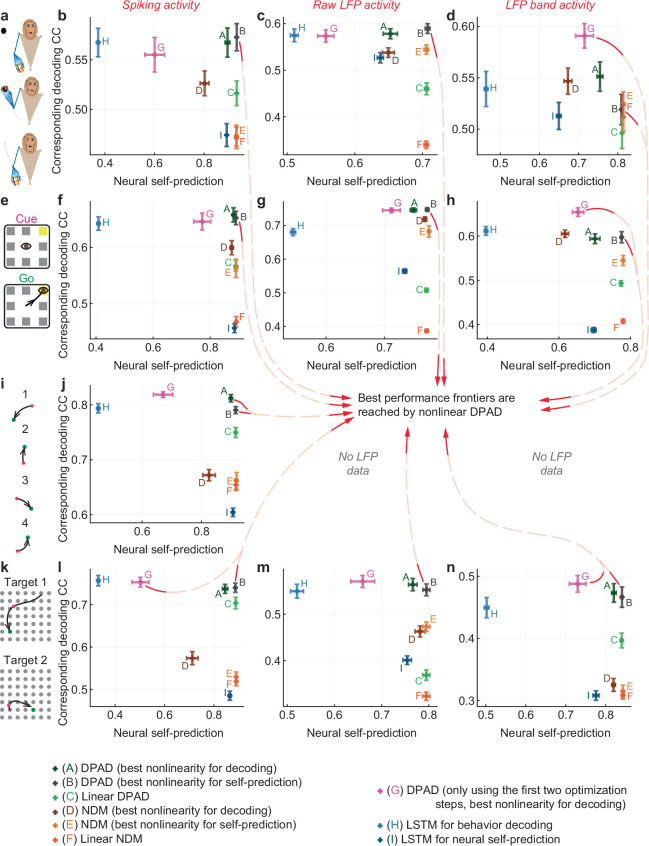
Across spiking and LFP neural modalities, DPAD is on the best performance frontier for neural-behavioral prediction unlike LSTMs, which are fitted to explain neural data or behavioral data. **a**, The 3D reach task. **b**, Cross-validated neural self-prediction accuracy achieved by each method versus the corresponding behavior decoding accuracy on the vertical axis. Latent state dimension for each method in each session and fold is chosen (among powers of 2 up to 128) as the smallest that reaches peak neural self-prediction in training data or reaches peak decoding in training data, whichever is larger (Methods). Pluses and whiskers are defined as in Fig. 3 (*N* = 35 session-folds). Note that DPAD considers an LSTM as a special case (Methods). Nevertheless, results are also shown for LSTM networks fitted to decode behavior from neural activity (that is, RNN decoders in Extended Data Table 1) or to predict the next time step of neural activity (self-prediction). Also, note that LSTM for behavior decoding (denoted by H) and DPAD when only using the first two optimization steps (denoted by G) dedicate all their latent states to behavior prediction, whereas other methods dedicate some or all latent states to neural self-prediction. Compared with all methods including these LSTM networks, DPAD always reaches the best performance frontier for predicting the neural-behavioral data whereas LSTM does not; this is partly due to the four-step optimization algorithm in DPAD that allows for overall neural-behavioral description rather than one or the other, and that prioritizes the learning of the behaviorally relevant neural dynamics. **c**, Same as **b** for raw LFP activity (*N* = 35 session-folds). **d**, Same as **b** for LFP band power activity (*N* = 35 session-folds). **e**-**h**, Same as **a**-**d** for the second dataset, with saccadic eye movements (*N* = 35 session-folds). **i**,**j**, Same as **a** and **b** for the third dataset, with sequential cursor reaches controlled via a 2D manipulandum (*N* = 15 session-folds). **k**-**n**, Same as **a**-**d** for the fourth dataset, with random grid virtual reality cursor reaches controlled via fingertip position (*N* = 35 session-folds). Results and conclusions are consistent across all datasets. Source data

Across all datasets and modalities, compared to nonlinear NDM or linear DPAD, nonlinear DPAD reached higher behavior decoding accuracy while also being as accurate or better in terms of neural self-prediction (Fig. 3, Extended Data Fig. 3 and Supplementary Fig. 4). Indeed, compared to these, DPAD was always on the best performance frontier for predicting the neural–behavioral data (Fig. 3 and Extended Data Fig. 3). Additionally, DPAD was always on the best performance frontier for predicting the neural–behavioral data compared to long short-term memory (LSTM) networks as well as a concurrently^44^ developed method with DPAD termed CEBRA^36^ on our four datasets (Fig. 4a–h) in addition to a fifth movement dataset^54^ analyzed in the CEBRA paper (Fig. 4i,j). These results suggest that DPAD provides a more accurate description for neural–behavioral data.

**Extended Data Fig. 4:**
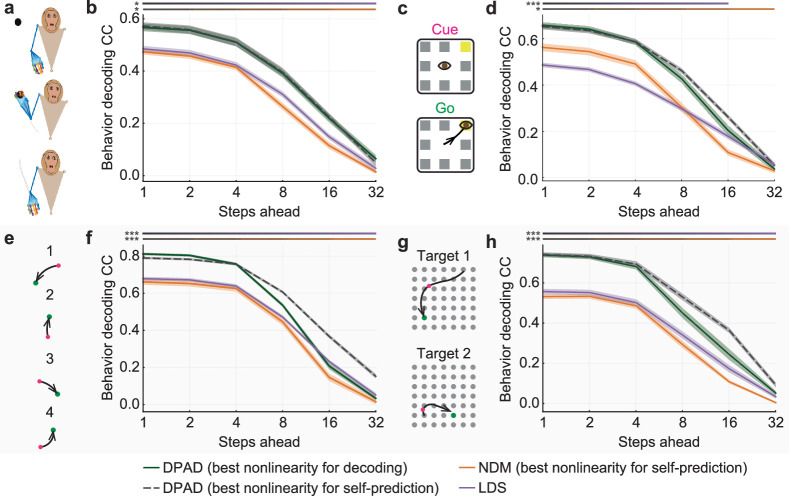
DPAD can also be used for multi-step-ahead forecasting of behavior. **a**, The 3D reach task. **b**, Cross-validated behavior decoding accuracy for various numbers of steps into the future. For *m*-step-ahead prediction, behavior at time step *k* is predicted using neural activity up to time step *k*−*m*. All models are taken from Fig. 3, without any retraining or finetuning, with *m*-step-ahead forecasting done by repeatedly (*m*−1 times) passing the neural predictions of the model as its neural observation in the next time step (Methods). Solid lines and shaded areas are defined as in Fig. 5b (*N* = 35 session-folds). Across the number of steps ahead, the statistical significance of a one-sided pairwise comparison between nonlinear DPAD vs nonlinear NDM is shown with the orange top horizontal line with p-value indicated by asterisks next to the line as defined in Fig. 2b (N = 35 session-folds). Similar pairwise comparison between nonlinear DPAD vs linear dynamical system (LDS) modeling is shown with the purple top horizontal line. **c**-**d**, Same as **a**-**b** for the second dataset, with saccadic eye movements (*N* = session-folds). **e**-**f**, Same as **a**-**b** for the third dataset, with sequential cursor reaches controlled via a 2D manipulandum (*N* = 15 session-folds). **g**-**h**, Same as **a**-**b** for the fourth dataset, with random grid virtual reality cursor reaches controlled via fingertip position (*N* = 35 session-folds).

Beyond one-step-ahead predictions, we next evaluated DPAD in terms of multistep-ahead prediction of neural–behavioral data, also known as forecasting. To do this, starting with one-step-ahead predictions (that is, *m* = 1), we pass *m*-step-ahead predictions of neural data using the learned models as the neural observation in the next time step to obtain (*m* + 1)-step-ahead predictions (Methods). Nonlinear DPAD was consistently better than nonlinear NDM and linear dynamical systems (LDS) modeling in multistep-ahead forecasting of behavior (Extended Data Fig. 4). For neural self-prediction, we used a naive predictor as a conservative forecasting baseline, which reflects how easy it is to predict the future in a model-free way purely based on the smoothness of neural data. DPAD significantly outperformed this baseline in terms of one-step-ahead and multistep-ahead neural self-predictions (Supplementary Fig. 5).

### Use-case 2: DPAD extracts behavior-predictive nonlinear transformations from raw LFP

We next used DPAD to compare the amount of nonlinearity in the neural–behavioral transformation across different neural modalities (Fig. 2 and Supplementary Fig. 3). To do so, we compared the gain in behavior decoding accuracy when going from linear to nonlinear DPAD modeling in each modality. In all datasets, raw LFP activity had the highest gain from nonlinearity in behavior decoding accuracy (Fig. 2c,f,k). Notably, using nonlinear DPAD, raw LFP reached more accurate behavior decoding than traditional LFP band powers in all tasks (Fig. 2b,e,j). In one dataset, raw LFP even significantly surpassed spiking activity in terms of behavior decoding accuracy (Fig. 2e). Note that computing LFP powers involves a prespecified nonreversible nonlinear transformation of raw LFP, which may be discarding important behaviorally relevant information that DPAD can uncover directly from raw LFP. Interestingly, linear dynamical modeling did worse for raw LFP than LFP powers in most tasks (compare linear DPAD for raw LFP versus LFP powers), suggesting that nonlinearity, captured by DPAD, was required for uncovering the extra behaviorally relevant information in raw LFP.

**Extended Data Fig. 5:**
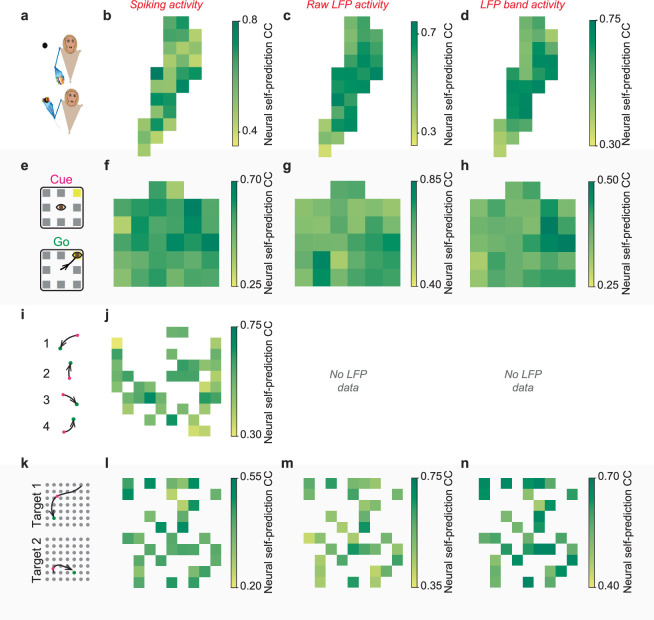
Neural self-prediction accuracy of nonlinear DPAD across recording electrodes for low-dimensional behaviorally relevant latent states. **a**, The 3D reach task. **b**, Average neural self-prediction correlation coefficient (CC) achieved by nonlinear DPAD for analyzed smoothed spiking activity is shown for each recording electrode (*N* = 35 session-folds; best nonlinearity for decoding). **c**, Same as **b** for modeling of raw LFP activity. **d**, Same as **b** for modeling of LFP band power activity. Here, prediction accuracy averaged across all 8 band powers (Methods) of a given recording electrode is shown for that electrode. **e-h**, Same **a**-**d** for the second dataset, with saccadic eye movements (*N* = 35 session-folds). For datasets with single-unit activity (Methods), spiking self-prediction of each electrode is averaged across the units associated with that electrode. **i**-**j**, Same as **a**,**b** for the third dataset, with sequential cursor reaches controlled via a 2D manipulandum (*N* = 15 session-folds). White areas are due to electrodes that did not have a neuron associated with them in the data. **k**-**n**, Same as **a**-**d** for the fourth dataset, with random grid virtual reality cursor reaches controlled via fingertip position (*N* = 35 session-folds). For all results, the latent state dimension was 16, and all these dimensions were learned using the first optimization step (that is, n_1_ = 16).

We next examined the spatial pattern of behaviorally relevant information across recording channels. For different channels, we compared the neural self-prediction of DPAD’s low-dimensional behaviorally relevant latent states (Extended Data Fig. 5). We computed the coefficient of variation (defined as standard deviation divided by mean) of the self-prediction over recording channels and found that the spatial distribution of behaviorally relevant information was less variable in raw LFP than spiking activity (*P* ≤ 0.00071, one-sided signed-rank test, *N* = 35 for all three datasets with LFP). This could suggest that raw LFPs reflect large-scale network-level behaviorally relevant computations, which are thus less variable within the same spatial brain area than spiking, which represents local, smaller-scale computations^55^.

### Use-case 3: DPAD enables behavior-predictive nonlinear dynamical dimensionality reduction

**Fig. 5:**
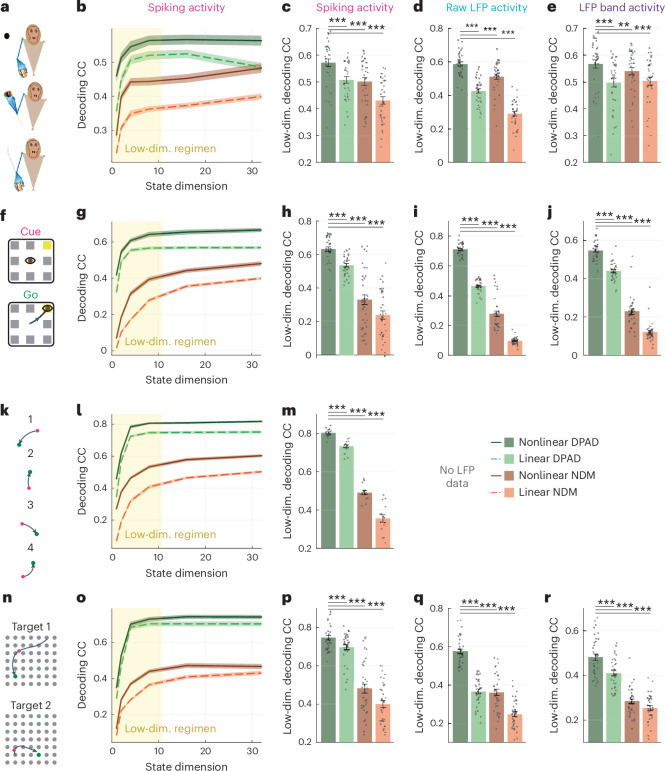
DPAD enables nonlinear and prioritized dynamical dimensionality reduction, thus learning more accurate models of behaviorally relevant neural dynamics with lower-dimensional latent states. **a**, The 3D reach task. **b**, Cross-validated decoding accuracy (CC) achieved by variations of linear/nonlinear DPAD/NDM for different latent state dimensions. For nonlinear DPAD/NDM, the nonlinearities are selected automatically based on the training data to maximize behavior decoding accuracy (flexible nonlinearity). Solid lines show the average across sessions and folds (*N* = 35 session-folds), and the shaded areas show the s.e.m.; Low-dim., low-dimensional. **c**, Decoding accuracy of nonlinear DPAD versus linear DPAD and nonlinear/linear NDM at the latent state dimension for which DPAD reaches within 5% of its peak decoding accuracy in the training data across all latent state dimensions. Bars, whiskers, dots and asterisks are defined as in Fig. 2b (*N* = 35 session-folds). **d**, Same as **c** for modeling of raw LFP (*N* = 35 session-folds). **e**, Same as **c** for modeling of LFP band power activity (*N* = 35 session-folds). **f**–**j**, Same as **a**–**e** for the second dataset with saccadic eye movements (*N* = 35 session-folds). **k**–**m**, Same as **a**–**c** for the third dataset, which did not include LFP data, with sequential cursor reaches controlled via a 2D manipulandum (*N* = 15 session-folds). **n**–**r**, Same as **a**–**e** for the fourth dataset, with random grid virtual reality cursor reaches controlled via fingertip position (*N* = 35 session-folds). For all DPAD variations, only the first two optimization steps were used in this figure (that is, *n*_1_ = *n*_*x*_) to only focus on learning behaviorally relevant neural dynamics in the dimensionality reduction regimen. Source data

**Extended Data Fig. 6:**
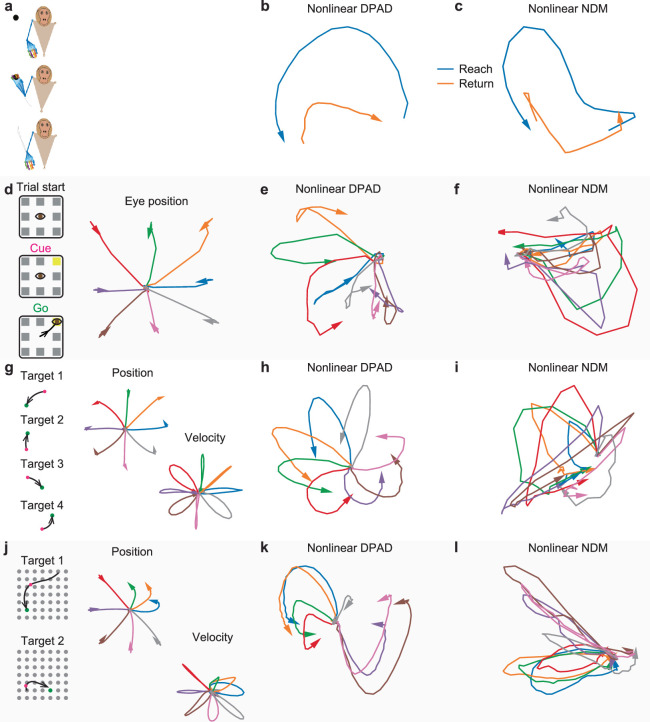
Nonlinear DPAD extracted distinct low dimensional latent states from neural activity for all datasets, which were more behaviorally relevant than those extracted using nonlinear NDM. **a**, The 3D reach task. **b**, The latent state trajectory for 2D states extracted from spiking activity using nonlinear DPAD, averaged across all reach and return epochs across sessions and folds. Here only optimization steps 1-2 of DPAD are used to just extract 2D behaviorally relevant states. **c**, Same as **b** for 2D states extracted using nonlinear NDM (special case of using just DPAD optimization steps 3-4). **d**, Saccadic eye movement task. Trials are averaged depending on the eye movement direction. **e**, The latent state trajectory for 2D states extracted using DPAD (extracted using optimizations steps 1-2), averaged across all trials of the same movement direction condition across sessions and folds. **f**, Same as **d** for 2D states extracted using nonlinear NDM. **g-i**, Same as **d**-**f** for the third dataset, with sequential cursor reaches controlled via a 2D manipulandum. **j**-**l**, Same as **d**-**f** for the fourth dataset, with random grid virtual reality cursor reaches controlled via fingertip position. Overall, in each dataset, latent states extracted by DPAD were clearly different for different behavior conditions in that dataset (**b**,**e**,**h**,**k**), whereas NDM’s extracted latent states did not as clearly dissociate different conditions (**c**,**f**,**i**,**l**). Of note, in the first dataset, DPAD revealed latent states with rotational dynamics that reversed direction during reach versus return epochs, which is consistent with the behavior roughly reversing direction. In contrast, NDM’s latent states showed rotational dynamics that did not reverse direction, thus were less congruent with behavior. In this first dataset, in our earlier work^6^, we had compared PSID and a subspace-based linear NDM method and, similar to **b** and **c** here, had found that only PSID uncovers reverse-directional rotational patterns across reach and return movement conditions. These results thus also complement our prior work^6^ by showing that even nonlinear NDM models may not uncover the distinct reverse-directional dynamics in this dataset, thus highlighting the need for dissociative and prioritized learning even in nonlinear modeling, as enabled by DPAD.

We next found that DPAD extracted latent states that were lower dimensional yet more behavior predictive than both nonlinear NDM and linear DPAD (Fig. 5). Specifically, we inspected the dimension required for nonlinear DPAD to reach almost (within 5% of) peak behavior decoding accuracy in each dataset (Fig. 5b,g,l,o). At this low latent state dimension, linear DPAD and nonlinear and linear NDM all achieved much lower behavior decoding accuracy than nonlinear DPAD across all neural modalities (Fig. 5c–e,h–j,m,p–r). The lower decoding accuracy of nonlinear NDM suggests that the dominant dynamics in spiking and LFP modalities can be unrelated to the modeled behavior. Thus, behaviorally relevant dynamics can be missed or confounded unless they are prioritized during nonlinear learning, as is done by DPAD. Moreover, we visualized the 2D latent state trajectories learned by each method (Extended Data Fig. 6). Consistent with the above results, DPAD extracted latent states from neural activity that were clearly different for different behavior/movement conditions (Extended Data Fig. 6b,e,h,k). In comparison, NDM extracted latent states that did not as clearly dissociate different conditions (Extended Data Fig. 6c,f,i,l). These results highlight the capability of DPAD for nonlinear dynamical dimensionality reduction in neural data while preserving behaviorally relevant neural dynamics.

**Extended Data Fig. 7:**
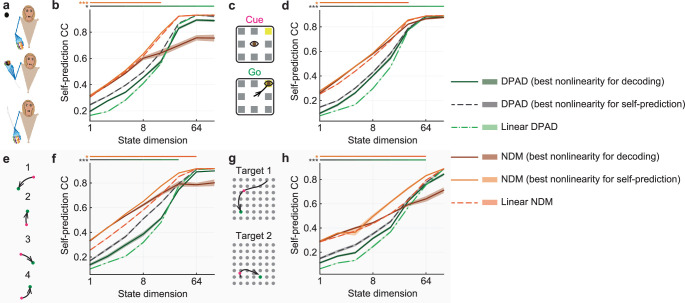
Neural self-prediction across latent state dimensions. **a**, The 3D reach task. **b**, Cross-validated neural self-prediction accuracy (CC) achieved by variations of nonlinear and linear DPAD/NDM, for different latent state dimensions. Solid lines and shaded areas are defined as in Fig. 5b (*N* = 35 session-folds). Across latent state dimensions, the statistical significance of a one-sided pairwise comparison between nonlinear DPAD/NDM (with best nonlinearity for self-prediction) vs linear DPAD/NDM is shown with a horizontal green/orange line with p-value indicated by asterisks next to the line as defined in Fig. 2b (*N* = 35 session-folds). **c**,**d**, Same as **a**,**b** for the second dataset, with saccadic eye movements (*N* = 35 session-folds). **e**,**f**, Same as **a**,**b** for the third dataset, with sequential cursor reaches controlled via a 2D manipulandum (*N* = 15 session-folds). **g**,**h** Same as **a**,**b** for the fourth dataset, with random grid virtual reality cursor reaches controlled via fingertip position (*N* = 35 session-folds). For all DPAD variations, the first 16 latent state dimensions are learned using the first two optimization steps and the remaining dimensions are learned using the last two optimization steps (that is, n_1_ = 16). As expected, at low state dimensions, DPAD’s latent states achieve higher behavior decoding (Fig. 5) but lower neural self-prediction than NDM because DPAD prioritizes the behaviorally relevant neural dynamics in these dimensions. However, by increasing the state dimension and utilizing optimization steps 3-4, DPAD can reach similar neural self-prediction to NDM while doing better in terms of behavior decoding (Fig. 3). Also, for low dimensional latent states, nonlinear DPAD/NDM consistently result in significantly more accurate neural self-prediction than linear DPAD/NDM. For high enough state dimensions, linear DPAD/NDM eventually reach similar neural self-prediction accuracy to nonlinear DPAD/NDM. Given that NDM solely aims to optimize neural self-prediction (irrespective of the relevance of neural dynamics to behavior), the latter result suggests that the overall neural dynamics can be approximated with linear dynamical models but only with high-dimensional latent states. Note that in contrast to neural self-prediction, behavior decoding of nonlinear DPAD is higher than linear DPAD even at high state dimensions (Fig. 3). Source data

Next, we found that at low dimensions, nonlinearity could improve the accuracy of both behavior decoding (Fig. 5b,g,l,o) and neural self-prediction (Extended Data Fig. 7). However, as the state dimension was increased, linear methods reached similar neural self-prediction performance as nonlinear methods across modalities (Fig. 3 and Extended Data Fig. 3). This was in contrast to behavior decoding, which benefited from nonlinearity regardless of how high the dimension was (Figs. 2 and 3).

### Use-case 4: DPAD localizes the nonlinearity in the neural–behavioral transformation

#### Numerical simulations validate DPAD’s localization

**Fig. 6:**
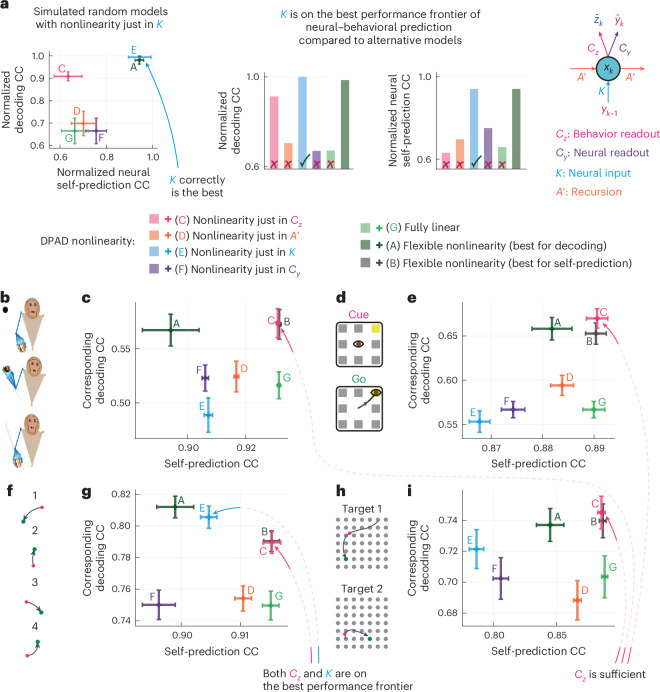
DPAD reveals that across our datasets, nonlinearities can be largely captured in the behavior readout of the model. **a**, The process of determining the origin of nonlinearity via hypothesis testing shown with an example simulation. Simulation results are taken from Extended Data Fig. 2b, and the origin is correctly identified as *K*. Pluses and whiskers are defined as in Fig. 3 (*N* = 20 random models). **b**, The 3D reach task. **c**, DPAD’s hypothesis testing. Cross-validated neural self-prediction accuracy (CC) for each nonlinearity and the corresponding decoding accuracy. DPAD variations that have only one nonlinear parameter (for example, *C*_*z*_) use a nonlinear neural network for that parameter and keep all other parameters linear. Linear and flexible nonlinear results are as in Fig. 3. Latent state dimension in each session and fold is chosen (among powers of 2 up to 128) as the smallest that reaches peak neural self-prediction in training data or reaches peak decoding in training data, whichever is larger (Methods). Pluses and whiskers are defined as in Fig. 3 (*N* = 35 session-folds). Annotated arrows indicate any individual nonlinearities that are on the best performance frontier compared to all other models. Results are shown for spiking activity here and for raw LFP and LFP power activity in Supplementary Fig. 6. **d**,**e**, Same as **b** and **c** for the second dataset with saccadic eye movements (*N* = 35 session-folds). **f**,**g**, Same as **b** and **c** for the third dataset, with sequential cursor reaches controlled via a 2D manipulandum (*N* = 15 session-folds). **h**,**i**, Same as **b** and **c** for the fourth dataset, with random grid virtual reality cursor reaches controlled via fingertip position (*N* = 35 session-folds). For all DPAD variations, the first 16 latent state dimensions are learned using the first two optimization steps, and the remaining dimensions are learned using the last two optimization steps (that is, *n*_1_ = 16). Source data

To demonstrate that DPAD can correctly find the origin of nonlinearity in the neural–behavioral transformation (Extended Data Fig. 2 and Supplementary Fig. 2), we simulated random models where only one of the parameters was set to a random nonlinear function (Methods). DPAD identifies a parameter as the origin if models with nonlinearity only in that parameter are on the best performance frontier when compared to alternative models, that is, models with nonlinearity in other parameters, models with flexible/full nonlinearity and fully linear models (Fig. 6a). DPAD enables this assessment due to (1) its flexible control over nonlinearities to train alternative models and (2) its simultaneous neural–behavioral modeling and evaluation (Methods). In all simulations, DPAD identified that the model with the correct nonlinearity origin was on the best performance frontier compared to alternative nonlinear models (Extended Data Fig. 2 and Supplementary Fig. 2), thus correctly revealing the origin of nonlinearity.

#### DPAD consistently localized nonlinearities in the behavior readout

Having validated the localization of nonlinearity in simulations, we used DPAD to find where in the model nonlinearities could be isolated to in our real datasets. We found that having the nonlinearity only in the behavior readout parameter *C*_*z*_ was largely sufficient for achieving high behavior decoding and neural self-prediction accuracies across all our datasets and modalities (Fig. 6b–i and Supplementary Fig. 6). First, for spiking activity, models with nonlinearity only in the behavior readout parameter *C*_*z*_ reached the best behavior decoding accuracy compared to models with other individual nonlinearities (Fig. 6c,e,i) while reaching almost the same decoding accuracy as fully nonlinear models (Fig. 6c,e,g,i). Second, these models with nonlinearity only in the behavior readout also reached a self-prediction accuracy that was unmatched by other types of individual nonlinearity (Fig. 6c,e,g,i). Overall, this meant that models with nonlinearity only in the behavior readout parameter *C*_*z*_ were always on the best performance frontier when compared to all other linear or nonlinear models (Fig. 6c,e,g,i). This result interestingly also held for both LFP modalities (Supplementary Fig. 6).

Consistent with the above localization results, DPAD with flexible nonlinearity also, very frequently, automatically selected models with nonlinearity in the behavior readout parameter (Supplementary Fig. 7). However, critically, this observation on its own cannot conclude that nonlinearities can be isolated in the behavior readout parameter. This is because in the flexible nonlinearity approach, parameters may be selected as nonlinear as long as this nonlinearity does not hurt the prediction accuracies, which does not imply that such nonlinearities are necessary (Methods); this is why we need the hypothesis testing procedure above (Fig. 6a). Of note, using an LSTM for the recursion parameter *A*′ is one of the nonlinearity options that is automatically considered in DPAD (Extended Data Fig. 3), but we found that LSTM was rarely selected in our datasets as the recursion dynamics in the flexible search over nonlinearities (Supplementary Fig. 7). Finally, note that fitting models with a nonlinear behavior readout via a post hoc nonlinear refitting of linear DPAD models (1) cannot identify the origin of nonlinearity in general (for example, other brain regions or tasks) and (2) even in our datasets resulted in significantly worse decoding than the same models being fitted end-to-end as done by nonlinear DPAD (*P* ≤ 0.0027, one-sided signed-rank test, *N* ≥ 15).

Together, these results highlight the application of DPAD in enabling investigations of nonlinear processing in neural computations underlying specific behaviors. DPAD’s machinery can not only fit fully nonlinear models but also provide evidence for the location in the model where the nonlinearity can be isolated (Discussion).

### Use-case 5: DPAD extends to noncontinuous and intermittent data

#### DPAD extends to intermittently sampled behavior observations

**Extended Data Fig. 8:**
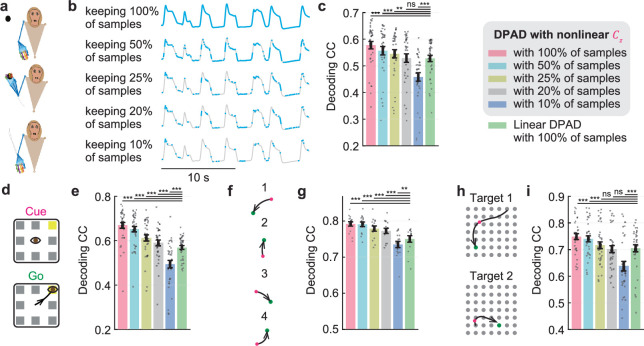
DPAD accurately learns the mapping from neural activity to behavior dynamics in all datasets even if behavioral samples are intermittently available in the training data. Nonlinear DPAD can perform accurately and better than linear DPAD even when as little as 20% of training behavior samples are kept. **a**, The 3D reach task. **b**, Examples are shown from one of the joints in the original behavior time series (light gray) and intermittently subsampled versions of it (cyan) where a subset of the time samples of the behavior time series are randomly chosen to be kept for use in training. In each subsampling, all dimensions of the behavior data are sampled together at the same time steps; this means that at any given time step, either all behavior dimensions are kept or all are dropped to emulate the realistic case with intermittent measurements. **c**, Cross-validated behavior decoding accuracy (CC) achieved by linear DPAD and by nonlinear DPAD with nonlinearity in the behavior readout parameter *C*_*z*_. For this nonlinear DPAD, we show the CC when trained with different percentage of behavior samples kept (that is, we emulate different rates of intermittent sampling). The state dimension in each session and fold is chosen (among powers of 2 up to 128) as the smallest that reaches peak decoding in training data. Bars, whiskers, dots, and asterisks are defined as in Fig. 2b (*N* = 35 session-folds). **d**,**e**, Same as **a**,**c** for the second dataset, with saccadic eye movements (*N* = 35 session-folds). **f**,**g**, Same as **a**,**c** for the third dataset, with sequential cursor reaches controlled via a 2D manipulandum (*N* = 15 session-folds). **h**,**i**, Same as **a**,**c** for the fourth dataset, with random grid virtual reality cursor reaches controlled via fingertip position (*N* = 35 session-folds). For all DPAD variations, the first 16 latent state dimensions are learned using the first two optimization steps and the remaining dimensions are learned using the last two optimization steps (that is, n_1_ = 16). Source data

**Extended Data Fig. 9:**
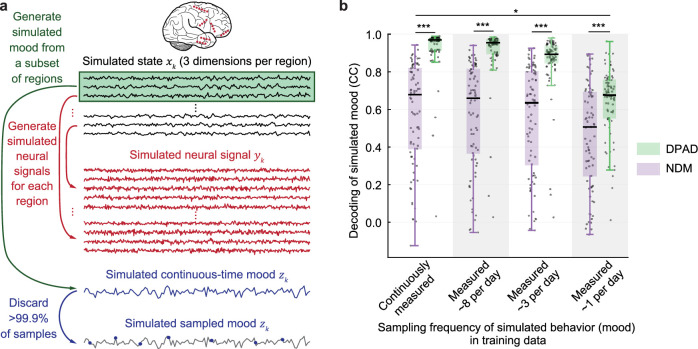
Simulations suggest that DPAD may be applicable with sparse sampling of behavior, for example with behavior being a self-reported mood survey value collected once per day. **a**, We simulated the application of decoding self-reported mood variations from neural signals^40,41^. Neural data is simulated based on linear models fitted to intracranial neural data recorded from epilepsy subjects. Each recorded region in each subject is simulated as a linear state-space model with a 3-dimensional latent state, with the same parameters as those fitted to neural recordings from that region. Simulated latent states from a subset of regions were linearly combined to generate a simulated mood signal (that is, biomarker). As the simulated models were linear, we used the linear versions of DPAD and NDM (NDM used the subspace identification method that we found does similarly to numerical optimization for linear models in Extended Data Fig. 1). We generated the equivalent of 3 weeks of intracranial recordings, which is on the order the time-duration of the real intracranial recordings. We then subsampled the simulated mood signal (behavior) to emulate intermittent behavioral measures such as mood surveys. **b**, Behavior decoding results in unseen simulated test data, across *N* = 87 simulated models, for different sampling rates of behavior in the training data. Box edges show the 25^th^ and 75^th^ percentiles, solid horizontal lines show the median, whiskers show the range of data, and dots show all data points (*N* = 87 simulated models). Asterisks are defined as in Fig. 2b. DPAD consistently outperformed NDM regardless of how sparse behavior measures were, even when these measures were available just once per day (*P* < 0.0005, one-sided signed-rank, *N* = 87).

DPAD also supports intermittently sampled behaviors (Methods)^56^, that is, when behavior is measured only during a subset of time steps. We first confirmed in numerical simulations with random models that DPAD correctly learns the model with intermittently sampled behavioral data (Supplementary Fig. 8). Next, in each of our neural datasets, we emulated intermittent sampling by randomly discarding up to 90% of behavior samples during learning. DPAD learned accurate nonlinear models even in this case (Extended Data Fig. 8). This capability is important, for example, in affective neuroscience or neuropsychiatry applications where the behavior consists of sparsely sampled momentary ecological assessments of mental states such as mood^40^. We next simulated a mood decoding application and found that with as low as one behavioral (for example, mood survey) sample per day, DPAD still outperformed NDM even when NDM had access to continuous behavior samples (Extended Data Fig. 9). These results suggest the potential utility of DPAD in such applications, although substantial future validation in data is needed^7,40–42^.

#### DPAD extends to noncontinuous-valued observations

**Fig. 7:**
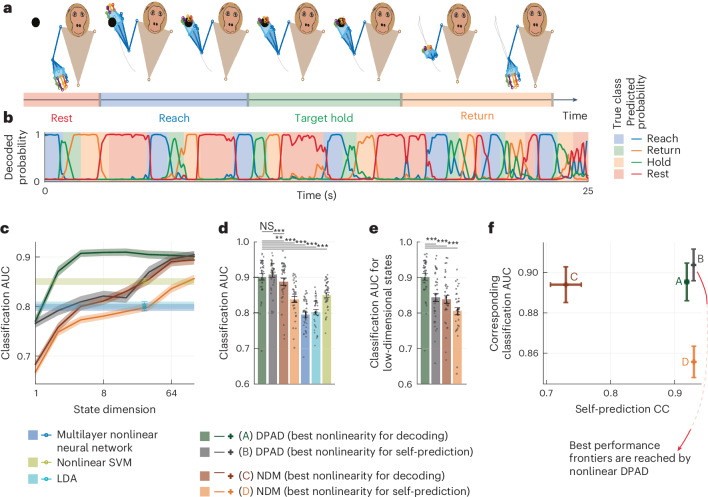
DPAD extends to modeling categorical behaviors. **a**, In the 3D reach dataset, we model spiking activity along with the epoch of the task as discrete behavioral data (Methods and Fig. 2a). The epochs/classes are (1) reaching toward the target, (2) holding the target, (3) returning to resting position and (4) resting until the next reach. **b**, DPAD’s predicted probability for each class is shown in a continuous segment of the test data. Most of the time, DPAD predicts the highest probability for the correct class. **c**, The cross-validated behavior classification performance, quantified as the area under curve (AUC) for the four-class classification, is shown for different methods at different latent state dimensions. Solid lines and shaded areas are defined as in Fig. 5b (*N* = 35 session-folds). AUC of 1 and 0.5 indicate perfect and chance-level classification, respectively. We include three nondynamic/static classification methods that map neural activity for a given time step to class label at the same time step (Extended Data Table 1): (1) multilayer neural network, (2) nonlinear support vector machine (SVM) and (3) linear discriminant analysis (LDA). **d**, Cross-validated behavior classification performance (AUC) achieved by each method when choosing the state dimension in each session and fold as the smallest that reaches peak classification performance in the training data among all state dimensions with that method (Methods). Bars, whiskers, dots and asterisks are defined as in Fig. 2b (*N* = 35 session-folds). **e**, Same as **d** when all methods use the same latent state dimension as DPAD (best nonlinearity for decoding) does in **d** (*N* = 35 session-folds). **c** and **e** show DPAD’s benefit for dimensionality reduction. **f**, Cross-validated neural self-prediction accuracy achieved by each method versus the corresponding behavior classification performance. Here, the latent state dimension for each method in each session and fold is chosen (among powers of 2 up to 128) as the smallest that reaches peak neural self-prediction in training data or reaches peak decoding in training data, whichever is larger (Methods). Pluses and whiskers are defined as in Fig. 3 (*N* = 35 session-folds). Source data

DPAD also extends to modeling of noncontinuous-valued (for example, categorical) behaviors (Methods). To demonstrate this, we modeled the transformation from neural activity to the momentary phase of the task in the 3D reach task: reach, hold, return or rest (Fig. 7). Compared to nonlinear NDM (which is dynamic) or nonlinear nondynamic methods such as support vector machines, DPAD more accurately predicted the task phase at each point in time (Fig. 7). This capability can extend the utility of DPAD to categorical behaviors such as decision choices in cognitive neuroscience^39^.

Finally, we applied DPAD to nonsmoothed spike counts, where we compared the results with two noncausal sequential autoencoder methods, termed LFADS^16^ and TNDM^18^ (Supplementary Fig. 9), both of which have Poisson observations that model nonsmoothed spike counts^16,18^. TNDM^18^, which was developed after LFADS^16^ and concurrently with our work^44,56^, adds behavioral terms to the objective function for a subset of latents but unlike DPAD does so with a mixed objective and thus does not completely dissociate or prioritize behaviorally relevant dynamics (Extended Data Table 1 and Supplementary Note 3). Compared to both LFADS and TNDM, DPAD remained on the best performance frontier for predicting the neural–behavioral data (Supplementary Fig. 9a) and more accurately predicted behavior using low-dimensional latent states (Supplementary Fig. 9b). Beyond this, TNDM and LFADS also have fundamental differences with DPAD and do not address some of DPAD’s use-cases (Discussion and Extended Data Table 1).

## Discussion

We developed DPAD for nonlinear dynamical modeling and investigation of neural dynamics underlying behavior. DPAD can dissociate the behaviorally relevant neural dynamics and prioritize their learning over other neural dynamics, enable hypothesis testing regarding the origin of nonlinearity in the neural–behavioral transformation and achieve causal decoding. DPAD enables prioritized dynamical dimensionality reduction by extracting lower-dimensional yet more behavior-predictive latent states from neural population activity and supports modeling noncontinuous-valued (for example, categorical) and intermittently sampled behavioral data. These attributes make DPAD suitable for diverse use-cases across neuroscience and neurotechnology, some of which we demonstrated here.

We found similar results for three neural modalities: spiking activity, LFP band powers and raw LFP. For all modalities, nonlinear DPAD more accurately learned the behaviorally relevant neural dynamics than linear DPAD and linear/nonlinear NDM as reflected in its better decoding while also reaching the best performance frontier when considering both behavior decoding and neural self-prediction. Notably, the raw LFP activity benefited the most from nonlinear modeling using DPAD and outperformed LFP powers in all tasks in terms of decoding. This suggests that automatic learning of nonlinear models from raw LFP using DPAD reveals behaviorally relevant information that may be discarded when extracting traditionally used features such as LFP band powers. Also, nonlinearity was necessary to recover the extra information in raw LFP, as, unlike DPAD modeling, linear dynamical modeling of raw LFP did not outperform that of LFP powers in most datasets. These results highlight another use-case of DPAD for automatic dynamic feature extraction from LFP data.

As another use-case, DPAD enabled an investigation of which element in the neural–behavioral transformation was nonlinear. Interestingly, consistently across our four movement-related datasets, DPAD models with nonlinearity only in the behavior readout performed similarly to fully nonlinear models, reaching the best performance frontier for predicting future behavior and neural data using past neural data. The consistency of this result across our datasets is interesting because, as demonstrated in simulations (Extended Data Fig. 2, Supplementary Fig. 2 and Fig. 6a), the detected origin of nonlinearity could have technically been in any one (or more) of the following four elements (Fig. 1a,b): neural input, recurrent dynamics and neural or behavior readouts, all of which were correctly localized in simulations (Extended Data Fig. 2 and Supplementary Fig. 2). Thus, the consistent localization results on our neural datasets provide evidence that across these four tasks, neural dynamics in these recorded cortical areas may be largely describable with linear dynamics of sufficiently high dimension, with additional nonlinearities introduced somewhere between the neural state and behavior. This finding may be consistent with (1) introduction of nonlinear processing along the downstream neuromuscular pathway that goes from the recorded cortical area to the measured behavior or any of the convergent inputs along this pathway^57–59^ or (2) cognition intervening nonlinearly between these latent neural states and behavior, for example, by implementing context-dependent computations^60^. This result illustrates how DPAD can provide new hypotheses and the machinery to test them in future experiments that would record from multiple additional brain regions (for example, both motor and cognitive regions) and use DPAD to model them together. Such analyses may narrow down or revise the origin of nonlinearity for the wider neural–behavioral measurement set; for example, the state dynamics may be found to be nonlinear once additional brain regions are added. Localization of nonlinearity could also guide the design of competitive deep learning architectures that are more flexible or easier to implement in neurotechnologies such as brain–computer interfaces^61^.

Interestingly, the behavior decoding aspect of the localization finding here is consistent with a prior study^22^ that explored the mapping of the motor cortex to an electromyogram (EMG) during a one-dimensional movement task with varying forces and found that a fully linear model was worse than a nonlinear EMG readout in decoding the EMG^22^. However, as our simulations show (Extended Data Fig. 2b and Fig. 6a), comparing a linear model to a model that has nonlinear behavior readout is not sufficient to conclude the origin of nonlinearity, and a stronger test is needed (see Fig. 6a for a counter example and details in Methods). Further, this previous study^22^ used a specific condition-dependent nonlinearity for behavior readout rather than a universal nonlinear function approximator that DPAD enables. Finally, to conclude localization, the model with that specific nonlinearity should perform similarly to fully nonlinear models; however, unlike our results, a fully nonlinear LSTM model in some cases appears to outperform models with nonlinear readout in this prior study (see Fig. 7a,b in ref. ^22^ versus Fig. 9c in ref. ^22^); it is unclear if this result is due to this prior study’s specific readout nonlinearity being suboptimal or to the nonlinear origin being different in its dataset^22^. DPAD can address such questions by (1) allowing for training and comparison of alternative models with different nonlinear origins and (2) enabling a general (versus specific) nonlinearity in model parameters.

When hypothesis testing about where in the model nonlinearity can be isolated to, it may be possible to equivalently explain the same data with multiple types of nonlinearities (for example, with either a nonlinear neural input or a nonlinear readout). Such nonidentifiability is a common limitation for latent models. However, when such equivalence exists, we expect all equivalent nonlinear models to have similar performance and thus lie on the best performance frontier. But this was not the case in our datasets. Instead, we found that the nonlinear behavior readout was in most cases the only individual nonlinear parameter on the best performance frontier, providing evidence that no other individual nonlinear parameter was as suitable in our datasets. Alternatively, the best model describing the data may require two or more of the four parameters to be nonlinear. But in our datasets, models with nonlinearity only in the behavior readout were always on the best performance frontier and could not be considerably outperformed by models with more than one nonlinearity (Fig. 6). Nevertheless, we note that ultimately our analysis simply provides evidence for one location of nonlinearity resulting in a better fit to data with a parsimonious model, but it does not rule out other possibilities for explaining the data. For example, one could reformulate a nonlinear readout model by adding latent states and representing the readout nonlinearity as a recursion nonlinearity for the additional states, although such an equivalent but less parsimonious model may need more data to be learned as accurately. Finally, we also note that our conclusions were based on the datasets and family of nonlinear models (recursive RNNs) considered here, and thus we cannot rule out different conclusions in other scenarios and/or brain regions. Nevertheless, by providing evidence for a nonlinearity configuration, DPAD can provide testable hypotheses for future experiments that record from more brain regions.

Sequential autoencoders, spearheaded by LFADS^16^, have been used to smooth single-trial neural activity^16^ without considering relevance to behavior, which is a distinct goal as we showed in comparison to PSID in our prior work^6^. Notably, another sequential autoencoder, termed TNDM, has been developed concurrently with our work^44,56^ that adds a behavior term to the optimization objective^18^. However, these approaches do not enable several of the use-cases of DPAD here. First, unlike DPAD’s four-step learning approach, TNDM and LFADS use a single learning step with a neural-only objective (LFADS)^16^ or a mixed neural–behavioral objective (TNDM)^18^ that does not fully prioritize the behaviorally relevant neural dynamics (Extended Data Table 1 and Supplementary Note 3). DPAD’s prioritization is important for accurate learning of behaviorally relevant neural dynamics and for preserving them in dimensionality reduction, as our results comparing DPAD to TNDM/LFADS suggest (Supplementary Fig. 9). Second, TNDM and LFADS^16,18^, like other prior works^16,18,20,23,24,26,61^, do not provide flexible nonlinearity or explore hypotheses regarding the origin of nonlinearities because they use fixed nonlinear network structures (use-case 4). Third, TNDM considers spiking activity and continuous behaviors^18^, whereas DPAD extends across diverse neural and behavioral modalities: spiking, raw LFP and LFP powers and continuous, categorical or intermittent behavioral modalities. Fourth, in contrast to these noncausal sequential autoencoders^16,18^ and some other nonlinear methods^8,14^, DPAD can process the test data causally and without expensive computations such as iterative expectation maximization^8,14^ or sampling and averaging^16,18^. This causal efficient processing is also important for real-time closed-loop brain–computer interfaces^62,63^. Of note, noncausal processing is also implemented in the DPAD code library as an option (Methods), although it is not shown in this work. Finally, unlike these prior methods^14,16,18^, DPAD does not require fixed-length trials or trial structure, making it suitable for modeling naturalistic behaviors^5^ and neural dynamics with trial-to-trial variability in the alignment to task events^64^.

Several methods can in some ways prioritize behaviorally relevant information while extracting latent embeddings from neural data but are distinct from DPAD in terms of goals and capabilities. One group includes nondynamic/static methods that do not explicitly model temporal dynamics^1^. These methods build linear maps (for example, as in demixed principal component analysis (dPCA)^34^) or nonlinear maps, such as convolutional maps in a concurrently^44^ developed method with DPAD named CEBRA^36^, to extract latent embeddings that can be guided by behavior either as a trial condition^34^ or indirectly as a contrastive loss^36^. These nondynamic mappings only use a single sample or a small fixed window around each sample of neural data to extract latent embeddings (Extended Data Table 1). By contrast, DPAD can recursively aggregate information from all past neural data by explicitly learning a model of temporal dynamics (recursion), which also enables forecasting unlike in static/nondynamic methods. These differences may be one reason why DPAD outperformed CEBRA in terms of neural–behavioral prediction (Fig. 4). Another approach is used by task aligned manifold estimation (TAME-GP)^9^, which uses a Gaussian process prior (as in Gaussian process factor analysis (GPFA)^14^) to expand the window of neural activity used for extracting the embedding into a complete trial. Unlike DPAD, methods with a Gaussian process prior have limited support for nonlinearity, often do not have closed-forms for inference and thus necessitate numerical optimization even for inference^9^ and often operate noncausally^9^. Finally, the above methods do not provide flexible nonlinearity or hypothesis testing to localize the nonlinearity.

Other prior works have used RNNs either causally^20,22–24,26^ or noncausally^16,18^, for example, for causal decoding of behavior from neural activity^20,22–24,26^. These works^20,22–24,26^ have similarities to the first step of DPAD’s four-step optimization (Supplementary Fig. 1a) in that the RNNs in these works learn dynamical models by solely optimizing behavior prediction. However, these works do not learn the mapping from the RNN latent states to neural activity, which is done in DPAD’s second optimization step to enable neural self-prediction (Supplementary Fig. 1a). In addition, unlike what the last two optimization steps in DPAD enable, these prior works do not model additional neural dynamics beyond those that decode behavior and thus do not dissociate the two types of neural dynamics (Extended Data Table 1). Finally, as noted earlier, these prior works^9,20,23,24,26,36,61^, similar to prior sequential autoencoders^16,18^, have fixed nonlinear network structures and thus cannot explore hypotheses regarding the origin of nonlinearities or flexibly learn the best nonlinear structure for the training data (Fig. 1c,d and Extended Data Table 1).

DPAD’s optimization objective functions are not convex, similar to most nonlinear deep learning methods. Thus, as usual with nonconvex optimizations, convergence to a global optimum is not guaranteed. Moreover, as with any method, quality and neural–behavioral prediction of the learned models depend on dataset properties such as signal-to-noise ratio. Thus, we compare alternative methods within each dataset, suggesting that (for example, Fig. 4) across the multiple datasets here, DPAD learns more accurate models of neural–behavioral data. However, models in other datasets/scenarios may not be as accurate.

Here, we focused on using DPAD to model the transformation of neural activity to behavior. DPAD can also be used to study the transformation between other signals. For example, when modeling data from multiple brain regions, one region can be taken as the primary signal (*y*_*k*_) and another as the secondary signal (*z*_*k*_) to dissociate their shared versus distinct dynamics. Alternatively, when modeling the brain response to electrical^7,41,42^ or sensory^41,65,66^ stimulation, one could take the primary signal (*y*_*k*_) to be the stimulation and the secondary signal (*z*_*k*_) to be neural activity to dissociate and predict neural dynamics that are driven by stimulation. Finally, one may apply DPAD to simultaneously recorded brain activity from two subjects as primary and secondary signals to find shared intersubject dynamics during social interactions.

## Methods

### Model formulation

Equation (1) simplifies the DPAD model by showing both of its RNN sections as one, but the general two-section form of the model is as follows:

2 $$\left\{\begin{array}{c}\left[\begin{array}{c}{x}_{k+1}^{\left(1\right)}\\ {x}_{k+1}^{\left(2\right)}\end{array}\right]=\left[\begin{array}{c}{{A}^{{\prime} }}^{\left(1\right)}\left({x}_{k}^{\left(1\right)}\right)\\ {{A}^{{\prime} }}^{\left(2\right)}\left({x}_{k}^{\left(2\right)}\right)\end{array}\right]+\left[\begin{array}{c}{K}^{\,\left(1\right)}\left(\;{y}_{k}\right)\\ {K}^{\,\left(2\right)}\left({y}_{k},{x}_{k+1}^{\left(1\right)}\right)\end{array}\right]\\ {y}_{k}={C}_{y}^{\,\left(1\right)}\left({x}_{k}^{\left(1\right)}\right)+{C}_{y}^{\,\left(2\right)}\left({x}_{k}^{\left(2\right)}\right)+{e}_{k}\\ {z}_{k}={C}_{z}^{\,\left(1\right)}\left({x}_{k}^{\left(1\right)}\right)+{C}_{z}^{\,\left(2\right)}\left({x}_{k}^{\left(2\right)}\right)+{\epsilon }_{k}\end{array}.\right.$$

This equation separates the latent states of Eq. (1) into the following two parts: ${x}_{k}^{\left(1\right)}\in {{\mathbb{R}}}^{{n}_{1}}$ denotes the latent states of the first RNN section that summarize the behaviorally relevant dynamics, and ${x}_{k}^{\left(2\right)}\in {{\mathbb{R}}}^{{n}_{2}}$, with ${n}_{2}={n}_{x}-{n}_{1}$, denotes those of the second RNN section that represent the other neural dynamics (Supplementary Fig. 1a). Here, *A*^′(1)^, *A*^′(2)^, *K*^(1)^, *K*^(2)^, ${C}_{y}^{\,\left(1\right)}$, ${C}_{y}^{\,\left(2\right)}$, ${C}_{z}^{\,\left(1\right)}$ and ${C}_{z}^{\,\left(2\right)}$ are multi-input–multi-output functions that parameterize the model, which we learn using a four-step numerical optimization formulation expanded on in the next section (Supplementary Fig. 1a). DPAD also supports learning the initial value of the latent states at time 0 (that is, ${x}_{0}^{\left(1\right)}$ and ${x}_{0}^{\left(2\right)}$) as a parameter, but in all analyses in this paper, the initial states are simply set to 0 given their minimal impact when modeling long data sequences. Each pair of superscripted parameters (for example, *A*^′(1)^ and *A*^′(2)^) in Eq. (2) is a dissociated version of the corresponding nonsuperscripted parameter in Eq. (1) (for example, *A*′). The computation graph for Eq. (2) is provided in Fig. 1b (and Supplementary Fig. 1a). In Eq. (2), the recursions for computing ${x}_{k}^{\left(1\right)}$ are not dependent on ${x}_{k}^{\left(2\right)}$, thus allowing the former to be computed without the latter. By contrast, ${x}_{k}^{\left(2\right)}$ can depend on ${x}_{k}^{\left(1\right)}$, and this dependence is modeled via *K*^(2)^ (see Supplementary Note 2). Note that such dependence of ${x}_{k}^{\left(2\right)}$ on ${x}_{k}^{\left(1\right)}$ via *K*^(2)^ does not introduce new dynamics to ${x}_{k}^{\left(2\right)}$ because it does not involve the recursion parameter *A*^′(2)^, which describes the dynamics of ${x}_{k}^{\left(2\right)}$. This two-section RNN formulation is mathematically motivated by equivalent representations of a dynamical system model in different bases and by the relation between the predictor and stochastic forms of dynamical systems (Supplementary Notes 1 and 2).

For the RNN formulated in Eq. (1) or (2), neural activity *y*_*k*_ constitutes the input, and predictions of neural and behavioral signals are the outputs (Fig. 1b) given by

3 $$\left\{\begin{array}{c}{\hat{y}}_{k}={C}_{y}\left({x}_{k}\right)\\ {\hat{z}}_{k}={C}_{z}\left({x}_{k}\right)\end{array}.\right.$$

Note that each *x*_*k*_ is estimated purely using all past *y*_*k*_ (that is, *y*_1_, …, *y*_*k* *–* 1_), so the predictions in Eq. (3) are one-step-ahead predictions of *y*_*k*_ and *z*_*k*_ using past neural observations (Supplementary Note 1). Once the model parameters are learned, the extraction of latent states *x*_*k*_ involves iteratively applying the first line from Eq. (2), and predicting behavior or neural activity involves applying Eq. (3) to the extracted *x*_*k*_. As such, by writing the nonlinear model in predictor form^67,68^ (Supplementary Note 1), we enable causal and computationally efficient prediction.

### Learning: four-step numerical optimization approach

#### Background

Unlike nondynamic models^1,34–36,69^, dynamical models explicitly model temporal evolution in time series data. Recent dynamical models have gone beyond linear or generalized linear dynamical models^2–7,70–81^ to incorporate switching linear^10–13^, locally linear^37^ or nonlinear^14–21,23,24,26,27,38,61,82–90^ dynamics, often using deep learning methods^25,91–94^. But these recent nonlinear/switching works do not aim to localize nonlinearity or allow for flexible nonlinearity and do not enable fully prioritized dissociation of behaviorally relevant neural dynamics because they either do not consider behavior in their learning objective at all^14,16,37,38,61,95,96^ or incorporate it with a mixed neural–behavioral objective^9,18,35,61^ (Extended Data Table 1).

In DPAD, we develop a four-step learning method for training our two-section RNN in Eq. (1) and extracting the latent states that (1) enables dissociation and prioritized learning of the behaviorally relevant neural dynamics in the nonlinear model, (2) allows for flexible modeling and localization of nonlinearities, (3) extends to data with diverse distributions and (4) does all this while also achieving causal decoding and being applicable to data both with and without a trial structure. DPAD is for nonlinear modeling, and its multistep learning approach, in each step, uses numerical optimization tools that are rooted in deep learning. Thus, DPAD is mathematically distinct from our prior PSID work for linear models, which is an analytical and linear technique. PSID is based on analytical linear algebraic projections rooted in control theory^6^, which are thus not extendable to nonlinear modeling or to non-Gaussian, noncontinuous or intermittently sampled data. Thus, even when we restrict DPAD to linear modeling as a special case, it is still mathematically different from PSID^6^.

#### Overview

To dissociate and prioritize the behaviorally relevant neural dynamics, we devise a four-step optimization approach for learning the two-section RNN model parameters (Supplementary Fig. 1a). This approach prioritizes the extraction and learning of the behaviorally relevant dynamics in the first two steps with states ${x}_{k}^{\left(1\right)}\in {{\mathbb{R}}}^{{n}_{1}}$ while also learning the rest of the neural dynamics in the last two steps with states ${x}_{k}^{\left(2\right)}\in {{\mathbb{R}}}^{{n}_{2}}$ and dissociating the two subtypes of dynamics. This prioritization is important for accurate learning of behaviorally relevant neural dynamics and is achieved because of the multistep learning approach; the earlier steps learn the behaviorally relevant dynamics first, that is, with priority, and then the subsequent steps learn the other neural dynamics later so that they do not mask or confound the behaviorally relevant dynamics. Importantly, each optimization step is independent of subsequent steps so all steps can be performed in order, with no need to iteratively repeat any step. We define the neural and behavioral prediction losses that are used in the optimization steps based on the negative log-likelihoods (NLLs) associated with the neural and behavior distributions, respectively. This approach benefits from the statistical foundation of maximum likelihood estimation and facilitates generalizability across behavioral distributions. We now expand on each of the four optimization steps for RNN training.

#### Optimization step 1

In the first two optimization steps (Supplementary Fig. 1a), the objective is to learn the behaviorally relevant latent states ${x}_{k}^{\left(1\right)}$ and their associated parameters. In the first optimization step, we learn the parameters *A*^′(1)^, ${C}_{z}^{\,\left(1\right)}$ and *K*^(1)^ of the RNN

4 $$\left\{\begin{array}{c}{x}_{k+1}^{\left(1\right)}={{A}^{{\prime} }}^{\left(1\right)}\left({x}_{k}^{\left(1\right)}\right)+{K}^{\,\left(1\right)}\left(\;{y}_{k}\right)\\ {z}_{k}={C}_{z}^{\,\left(1\right)}\left({x}_{k}^{\left(1\right)}\right)+{\epsilon }_{k}\end{array}\right.$$

and estimate its latent state ${x}_{k}^{\left(1\right)}$ while minimizing the NLL of the behavior *z*_*k*_ given by ${x}_{k}^{\left(1\right)}$. For continuous-valued (Gaussian) behavioral data, we minimize the following sum of squared prediction error^69,97^ given by

5 $${L}_{z}^{(1)}=\sum _{k}{\left\Vert {z}_{k}-{\hat{z}}_{k}\right\Vert }_{2}^{2}=\sum _{k}{\left\Vert {z}_{k}-{C}_{z}^{\,(1)}({x}_{k}^{(1)})\right\Vert }_{2}^{2}$$

where the sum is over all available samples of behavior *z*_*k*_, and ${\Vert .\Vert }_{2}$ indicates the two-norm operator. This objective, which is typically used when fitting models to continuous-valued data^69,97^, is proportional to the Gaussian NLL if we assume isotropic Gaussian residuals (that is, ∑_𝜖_ = σ_𝜖_*I*)^69,97^. If desired, a general nonisotropic residual covariance ∑_𝜖_ can be empirically computed from model residuals after the above optimization is solved (see Learning noise statistics), although having ∑_𝜖_ is mainly useful for simulating new data and is not needed when using the learned model for inference. Similarly, in the subsequent optimization steps detailed later, the same points hold regarding how the appropriate mean squared error used for continuous-valued data is proportional to the Gaussian NLL if we assume isotropic Gaussian residuals and how the residual covariance can be computed empirically after the optimization if desired.

#### Optimization step 2

The second optimization step uses the extracted latent state ${x}_{k}^{\left(1\right)}$ from the RNN and fits the parameter ${C}_{y}^{\left(1\right)}$ in

6 $${y}_{k}={C}_{y}^{\,\left(1\right)}\left({x}_{k}^{\left(1\right)}\right)+{e}_{k}$$

while minimizing the NLL of the neural activity *y*_*k*_ given by ${x}_{k}^{(1)}$. For continuous-valued (Gaussian) neural activity *y*_*k*_, we minimize the following sum of squared prediction error^69^:

7 $${L}_{y}^{(1)}=\sum _{k}{\left\Vert\, {y}_{k}-\hat{y}_{k}\right\Vert }_{2}^{2}=\sum _{k}{\left\Vert\, {y}_{k}-{C}_{y}^{\,(1)}({x}_{k}^{(1)})\right\Vert }_{2}^{2},$$

where the sum is over all available samples of *y*_*k*_. Optimization steps 1 and 2 conclude the prioritized extraction and modeling of behaviorally relevant latent states ${x}_{k}^{(1)}$ (Fig. 1b) and the learning of the first section of the RNN model (Supplementary Fig. 1a).

#### Optimization step 3

In optimization steps 3 and 4 (Supplementary Fig. 1a), the objective is to learn any additional dynamics in neural activity that are not learned in the first two optimization steps, that is, ${x}_{k}^{\left(2\right)}$ and the associated parameters. To do so, in the third optimization step, we learn the parameters *A*^′(2)^, ${C}_{y}^{\,\left(2\right)}$ and *K*^(2)^ of the RNN

8 $$\left\{\begin{array}{c}{x}_{k+1}^{\left(2\right)}={{A}^{{\prime} }}^{\left(2\right)}\left({x}_{k}^{\left(2\right)}\right)+{K}^{\,\left(2\right)}\left({y}_{k},{x}_{k+1}^{\left(1\right)}\right)\\ {y}_{k}^{{\prime} }={C}_{y}^{\,\left(2\right)}\left({x}_{k}^{\left(2\right)}\right)+{e}_{k}^{{\prime} }\end{array}\right.$$

and estimate its latent state ${x}_{k}^{\left(2\right)}$ while minimizing the aggregate NLL of *y*_*k*_ given both latent states, that is, by also taking into account the NLL obtained from step 2 via the ${C}_{y}^{\,\left(1\right)}\left({x}_{k}^{\left(1\right)}\right)$ term in Eq. (6). The notations ${y}_{k}^{{\prime} }$ and ${e}_{k}^{{\prime} }$ in the second line of Eq. (8) signify the fact that it is not *y*_*k*_ that is predicted by the RNN of Eq. (8), rather it is the yet unpredicted parts of *y*_*k*_ (that is, unpredicted after extracting ${x}_{k}^{(1)}$) that are being predicted. In the case of continuous-valued (Gaussian) neural activity *y*_*k*_, we minimize the following loss:

9 $${L}_{y}^{(2)}=\sum _{k}{\left\Vert\, {y}_{k}-{C}_{y}^{\,(1)}\left({x}_{k}^{(1)}\right)-{C}_{y}^{\,(2)}\left({x}_{k}^{(2)}\right)\right\Vert }_{2}^{2},$$

where the sum is over all available samples of *y*_*k*_. Note that in the continuous-valued (Gaussian) case, this loss is equivalent to minimizing the error in predicting the residual neural activity given by ${y}_{k}-{C}_{y}^{\,\left(1\right)}\left({x}_{k}^{\left(1\right)}\right)$ and is computed using the previously learned parameter ${C}_{y}^{\,\left(1\right)}$ and the previously extracted states ${x}_{k}^{\left(1\right)}$ in steps 1 and 2. Also, the input to the RNN in Eq. (8) includes both *y*_*k*_ and the extracted ${x}_{k+1}^{\left(1\right)}$ from optimization step 1. The above shows how the optimization steps are appropriately linked together to compute the aggregate likelihoods.

#### Optimization step 4

If we assume that the second set of states ${x}_{k}^{\left(2\right)}$ do not contain any information about behavior, we could stop the modeling. However, this may not be the case if the dimension of the states extracted in the first optimization step (that is, *n*_1_) is selected to be very small such that some behaviorally relevant neural dynamics are not learned in the first step. To be robust to such selections of *n*_1_, we can use another final numerical optimization to determine based on the data whether and how ${x}_{k}^{\left(2\right)}$ should affect behavior prediction. Thus, a fourth optimization step uses the extracted latent state in optimization steps 1 and 3 and fits *C*_*z*_ in

10 $${z}_{k}={C}_{z}\left({x}_{k}^{\left(1\right)},{x}_{k}^{\left(2\right)}\right)+{\epsilon }_{k}$$

while minimizing the negative log-likelihood of behavior given both latent states. In the case of continuous-valued (Gaussian) behavior *z*_*k*_, we minimize the following loss:

11 $${L}_{z}^{(2)}=\sum_{k}{\left\Vert {z}_{k}-\hat{z}_{k}\right\Vert}_{2}^{2}=\sum _{k}{\left\Vert {z}_{k}-{C}_{z}({x}_{k}^{(1)},{x}_{k}^{(2)})\right\Vert }_{2}^{2}.$$

The parameter *C*_*z*_ that is learned in this optimization step will replace both ${C}_{z}^{\,\left(1\right)}$ and ${C}_{z}^{\,\left(2\right)}$ in Eq. (2). Optionally, in a final optimization step, a similar nonlinear mapping from ${x}_{k}^{\left(1\right)}$ and ${x}_{k}^{\left(2\right)}$ can also be learned, this time to predict *y*_*k*_, which allows DPAD to support nonlinear interactions of ${x}_{k}^{\left(1\right)}$ and ${x}_{k}^{\left(2\right)}$ in predicting neural activity. In this case, the resulting learned *C*_*y*_ parameter will replace both ${C}_{y}^{\,\left(1\right)}$ and ${C}_{y}^{\,\left(2\right)}$ in Eq. (2). This concludes the learning of both model sections (Supplementary Fig. 1a) and all model parameters in Eq. (2).

In this work, when optimization steps 1 and 3 are both used to extract the latent states (that is, when 0 < *n*_1_ < *n*_*x*_), we do not perform the additional fourth optimization step in Eq. (10), and the prediction of behavior is done solely using the ${x}_{k}^{\left(1\right)}$ states extracted in the first optimization step. Note that DPAD can also cover NDM as a special case if we only use the third optimization step to extract the states (that is, *n*_1_ = 0, in which case the first two steps are not needed). In this case, we use the fourth optimization step to learn *C*_*z*_, which is the mapping from the latent states to behavior. Also, in this case, we simply have a unified state *x*_*k*_ as there is no dissociation in NDM, and the only goal is to extract states that predict neural activity accurately.

#### Additional generalizations of state dynamics

Finally, the first lines of Eqs. (4) and (8) can also be written more generally as

12 $${x}_{k+1}^{\left(1\right)}={{A}^{{\prime} {\prime} }}^{\left(1\right)}\left({x}_{k}^{\left(1\right)},{y}_{k}\right)$$

and

13 $${x}_{k+1}^{\left(2\right)}={{A}^{{\prime} {\prime} }}^{\left(2\right)}\left({x}_{k}^{\left(2\right)},{y}_{k},{x}_{k+1}^{\left(1\right)}\right),$$

where instead of an additive relation between the two terms of the righthand side, both terms are combined in nonlinear functions ${{A}^{{\prime} {\prime} }}^{\left(1\right)}$ and ${{A}^{{\prime} {\prime} }}^{\left(2\right)}$, which as a special case can still learn the additive relation in Eqs. (4) and (8). Whenever both the state recursion *A* and neural input *K* parameters (with the appropriate superscripts) are specified to be nonlinear, we use the more general architecture in Eqs. (12) and (13), and if any one of *A* or *K* or both are linear, we use Eqs. (4) and (8).

As another option, both RNN sections can be made bidirectional, which enables noncausal prediction for DPAD by using future data in addition to past data, with the goal of improving prediction, especially in datasets with stereotypical trials. Although this option is not reported in this work, it is implemented and available for use in DPAD’s public code library.

#### Learning noise statistics

Once the learning is complete, we also compute the covariances of the neural and behavior residual time series *e*_*k*_ and 𝜖_*k*_ as ∑_e_ and ∑_𝜖_, respectively. This allows the learned model in Eq. (1) to be usable for generating new simulated data. This application is not the focus of this work, but an explanation of it is provided in Numerical simulations.

#### Regularization

Adding norm 1 or norm 2 regularization for any set of parameters and the option to automatically select the regularization weight with inner cross-validation is implemented in the DPAD code. However, we did not use regularization in any of the analyses presented here.

#### Forecasting

DPAD also enables the capability to predict neural–behavioral data more than one time step into the future. To obtain two-step-ahead prediction, we pass the one-step-ahead neural predictions of the model as neural observations into it. This allows us to perform one state update iteration, that is, line 1 of Eq. (2), with *y*_*k*_ being replaced with ${\hat{y}}_{k}$ from Eq. (3). Repeating this procedure *m* times gives the (*m* + 1)-step-ahead prediction of the latent state and neural–behavioral data.

### Extending to intermittently measured behaviors

We also extend DPAD to modeling intermittently measured behavior time series (Extended Data Figs. 8 and 9 and Supplementary Fig. 8). To do so, when forming the behavior loss (Eqs. (5) and (11)), we only compute the loss on samples where the behavior is measured and solve the optimization with this loss.

### Extending to noncontinuous-valued data observations

We can also extend DPAD to noncontinuous-valued (non-Gaussian) observations by devising modified loss functions and observation models. Here, we demonstrate this extension for categorical behavioral observations, for example, discrete choices or epochs/phases during a task (Fig. 7). A similar approach could be used in the future to model other non-Gaussian behaviors and non-Gaussian (for example, Poisson) neural modalities, as shown in a thesis^56^.

To model categorical behaviors, we devise a new behavior observation model for DPAD by making three changes. First, we change the behavior loss (Eqs. (5) and (11)) to the NLL of a categorical distribution, which we implement using the dedicated class in the TensorFlow library (that is, tf.keras.losses.CategoricalCrossentropy). Second, we change the behavior readout parameter *C*_*z*_ to have an output dimension of *n*_z_ × *n*_*c*_ instead of *n*_*z*_, where *n*_*c*_ denotes the number of behavior categories or classes. Third, we apply Softmax normalization (Eq. (14)) to the output of the behavior readout parameter *C*_*z*_ to ensure that for each of the *n*_*z*_ behavior dimensions, the predicted probabilities for all the *n*_*c*_ classes add up to 1 so that they represent valid probability mass functions. Softmax normalization can be written as

14 $${p}_{k}^{\left(m,n\right)}=\frac{\exp \left({l}_{k}^{\,\left(m,n\right)}\right)}{{\sum }_{i=1}^{{n}_{c}}\exp \left({l}_{k}^{\,\left(m,i\right)}\right)},$$

where ${l}_{k}\in {{\mathbb{R}}}^{{n}_{z}\times {n}_{c}}$ is the output of *C*_*z*_ at time *k*, and the superscript ^(*m*,*n*)^ denotes the element of *l*_*k*_ associated with the behavior dimension *m* and the class/category number *n*. With these changes, we obtain a new RNN architecture with categorical behavioral outputs. We then learn this new RNN architecture with DPAD’s four-step prioritized optimization approach as before but now incorporating the modified NLL losses for categorical data. Together, with these changes, DPAD extends to modeling categorical behavioral measurements.

### Behavior decoding and neural self-prediction metrics and performance frontier

#### Cross-validation

To evaluate the learning, we perform a cross-validation with five folds (unless otherwise noted). We cut the data from the recording session into five equal continuous segments, leave these segments out one by one as the test data and train the model only using the data in the remaining segments. Once the model is trained using the neural and behavior training data, we pass the neural test data to the model to get the latent states in the test data using the first line of Eq. (1) (or Eq. (2), equivalently). We then pass the extracted latent states to Eq. (3) to get the one-step-ahead prediction of the behavior and neural test data, which we refer to as behavior decoding and neural self-prediction, respectively. Note that only past neural data are used to get the behavior and neural predictions. Also, the behavior test data are never used in predictions. Given the predicted behavior and neural time series, we compute the CC between each dimension of these time series and the actual behavior and neural test time series. We then take the mean of CC across dimensions of behavior and neural data to get one final cross-validated CC value for behavior decoding and one final CC value for neural self-prediction in each cross-validation fold.

#### Selection of the latent state dimension

We often need to select a latent state dimension to report an overall behavior decoding and/or neural self-prediction accuracy for each model/method (for example, Figs. 2–7). By latent state dimension, we always refer to the total latent state dimension of the model, that is, *n*_*x*_. For DPAD, unless otherwise noted, we always used *n*_1_ = 16 to extract the first 16 latent state dimensions (or all latent state dimensions when *n*_*x*_ ≤ 16) using steps 1 and 2 and any remaining dimensions using steps 3 and 4. We chose *n*_1_ = 16 because dedicating more, even all, latent state dimensions to behavior prediction only minimally improved it across datasets and neural modalities. For all methods, to select a state dimension *n*_*x*_, in each cross-validation fold, we fit models with latent state dimensions 1, 2, 4, 16,…and 128 (powers of 2 from 1 to 128) and select one of these models based on their decoding and neural self-prediction accuracies within the training data of that fold. We then report the decoding/self-prediction of this selected model computed in the test data of that fold. Our goal is often to select a model that simultaneously explains behavior and neural data well. For this goal, we pick the state dimension that reaches the peak neural self-prediction in the training data or the state dimension that reaches the peak behavior decoding in the training data, whichever is larger; we then report both the neural self-prediction and the corresponding behavior decoding accuracy of the same model with the selected state dimension in the test data (Figs. 3–4, 6 and 7f, Extended Data Figs. 3 and 4 and Supplementary Figs. 4–7 and 9). Alternatively, for all methods, when our goal is to find models that solely aim to optimize behavior prediction, we report the cross-validated prediction performances for the smallest state dimension that reaches peak behavior decoding in training data (Figs. 2, 5 and 7d, Extended Data Fig. 8 and Supplementary Fig. 3). We emphasize that in all cases, the reported performances are always computed in the test data of the cross-validation fold, which is not used for any other purpose such as model fitting or selection of the state dimension.

#### Performance frontier

When comparing a group of alternative models, we use the term ‘performance frontier’ to describe the best performances reached by models that in every comparison with any alternative model are in some sense better than or at least comparable to the alternative model. More precisely, when comparing a group ${\mathcal{M}}$ of models, model ${\mathcal{A}}\in {\mathcal{M}}$ will be described as reaching the best performance frontier when compared to every other model ${\mathcal{B}}{\mathscr{\in }}{\mathcal{M}}$, ${\mathcal{A}}$ is significantly better than ${\mathcal{B}}$ in behavior decoding or in neural self-prediction or is comparable to ${\mathcal{B}}$ in both. Note that ${\mathcal{A}}$ may be better than some model ${{\mathcal{B}}}_{1}\in {\mathcal{M}}$ in decoding while being better than another model ${{\mathcal{B}}}_{2}\in {\mathcal{M}}$ in self-prediction; nevertheless ${\mathcal{A}}$ will be on the frontier as long as in every comparison one of the following conditions hold: (1) there is at least one measure for which ${\mathcal{A}}$ is more performant and (2) ${\mathcal{A}}$ is at least equally performant in both measures. To avoid exclusion of models from the best performance frontier due to very minimal performance differences, in this analysis, we only declare a difference in performance significant if in addition to resulting in *P* ≤ 0.05 in a one-sided signed-rank test there is also at least 1% relative difference in the mean performance measures.

### DPAD with flexible nonlinearity: automatic determination of appropriate nonlinearity

#### Fine-grained control over nonlinearities

Each parameter in the DPAD model represents an operation in the computation graph of DPAD (Fig. 1b and Supplementary Fig. 1a). We solve the numerical optimizations involved in model learning in each step of our multistep learning via standard stochastic gradient descent^43^, which remains applicable for any modification of the computation graph that remains acyclic. Thus, the operation associated with each model parameter (for example, *A*′, *K*, *C*_*y*_ and *C*_*z*_) can be replaced with any multilayer neural network with an arbitrary number of hidden units and layers (Supplementary Fig. 1c), and the model remains trainable with the same approach. Having no hidden layers implements the special case of a linear mapping (Supplementary Fig. 1b). Of course, given that the training data are finite, the typical trade-off between model capacity and generalization error remains^69^. Given that neural networks can approximate any continuous function (with a compact domain)^98^, replacing model parameters with neural networks should have the capacity to learn any nonlinear function in their place^99–101^. The resulting RNN in Eq. (1) can in turn approximate any state-space dynamics (under mild conditions)^102^. In this work, for nonlinear parameters, we use multilayer feed-forward networks with one or two hidden layers, each with 64 or 128 units. For all hidden layers, we always use a rectified linear unit (ReLU) nonlinear activation (Supplementary Fig. 1c). Finally, when making a parameter (for example, *C*_*z*_) nonlinear, we always do so for that parameter in both sections of the RNN (for example, both ${C}_{z}^{\,\left(1\right)}$ and ${C}_{z}^{\,\left(2\right)}$; see Supplementary Fig. 1a) and using the same feed-forward network structure. Given that no existing RNN implementation allowed individual RNN elements to be independently set to arbitrary multilayer neural networks, we developed a custom TensorFlow RNN cell to implement the RNNs in DPAD (Eqs. (4) and (8)). We used the Adam optimizer to implement gradient descent for all optimization steps^43^. We continued each optimization for up to 2,500 epochs but stopped earlier if the objective function did not improve in three consecutive epochs (convergence criteria).

#### Automatic selection of nonlinearity settings

We devise a procedure for automatically determining the most suitable combination of nonlinearities for the data, which we refer to as DPAD with flexible nonlinearity. In this procedure, for each cross-validation fold in each recording session of each dataset, we try a series of nonlinearities within the training data and select one based on an inner cross-validation within the training data (Fig. 1d). Specifically, we consider the following options for the nonlinearity. First, each of the four main parameters (that is, *A*′, *K*, *C*_*y*_ and *C*_*z*_) can be linear or nonlinear, resulting in 16 cases (that is, 2^4^). In cases with nonlinearity, we consider four network structures for the parameters, that is, having one or two hidden layers and having 64 or 128 units in each hidden layer (Supplementary Fig. 1c), resulting in 61 cases (that is, 15 × 4 + 1, where 1 is for the fully linear model) overall. Finally, specifically for the recursion parameter *A*′, we also consider modeling it as an LSTM, with the other parameters still having the same nonlinearity options as before, resulting in another 29 cases for when this LSTM recursion is used (that is, 7 × 4 + 1, where 1 is for the case where the other three model parameters are all linear), bringing the total number of considered cases to 90. For each of these 90 considered linear or nonlinear architectures, we perform a twofold inner cross-validation within the training data to compute an estimate of the behavior decoding and neural self-prediction of each architecture using the training data. Note that although this process for automatic selection of nonlinearities is computationally expensive, it is parallelizable because each candidate model can be fitted independently on a different processor. Once all candidate architectures are fitted and evaluated within the training data, we select one final architecture purely based on training data to be used for that cross-validation fold based on one of the following two criteria: (1) decoding focused: pick the architecture with the best neural self-prediction in training data among all those that reach within 1 s.e.m. of the best behavior decoding; or (2) self-prediction focused: pick the architecture with the best behavior decoding in training data among all those that reach within 1 s.e.m. of the best neural self-prediction. The first criterion prioritizes good behavior decoding in the selection, and the second criterion prioritizes good neural self-prediction. Note that these two criteria are used when selecting among different already-learned models with different nonlinearities and thus are independent of the four internal objective functions used in learning the parameters for a given model with the four-step optimization approach (Supplementary Fig. 1a). For example, in the first optimization step of DPAD, model parameters are always learned to optimize behavior decoding (Eq. (5)). But once the four-step optimization is concluded and different models (with different combinations of nonlinearities) are learned, we can then select among these already-learned models based on either neural self-prediction or behavior decoding. Thus, whenever neural self-prediction is also of interest, we report the results for flexible nonlinearity based on both model selection criteria (for example, Figs. 3, 4 and 6).

#### Localization of nonlinearities

DPAD enables an inspection of where nonlinearities can be localized to by providing two capabilities, without either of which the origin of nonlinearities may be incorrectly found. As the first capability, DPAD can train alternative models with different individual nonlinearities and then compare these alternative nonlinear models not only with a fully linear model but also with each other and with fully nonlinear models (that is, flexible nonlinearity). Indeed, our simulations showed that simply comparing a linear model to a model with nonlinearity in a given parameter may incorrectly identify the origin of nonlinearity (Extended Data Fig. 2b and Fig. 6a). For example, in Fig. 6a, although the nonlinearity is just in the neural input parameter, a linear model does worse than a model with a nonlinear behavior readout parameter. Thus, just a comparison of the latter model to a linear model would incorrectly find the origin of nonlinearity to be the behavior readout. This issue is avoided in DPAD because it can also train a model with the neural input being nonlinear, thus finding it to be more predictive than models with any other individual nonlinearity and as predictive as a fully nonlinear model (Fig. 6a). As the second capability, DPAD can compare alternative nonlinear models in terms of overall neural–behavioral prediction rather than either behavior decoding or neural prediction alone. Indeed, our simulations showed that comparing the models in terms of just behavior decoding (Extended Data Fig. 2d,f) or just neural self-prediction (Extended Data Fig. 2d,h) may lead to incorrect conclusions about the origin of nonlinearities; this is because a model with the incorrect origin may be equivalent in one of these metrics to the one with the correct origin. DPAD avoids this problem by jointly evaluating both neural–behavioral metrics. Here, when comparing models with nonlinearity in different individual parameters for localization purposes (for example, Fig. 6), we only consider one network architecture for the nonlinearity, that is, having one hidden layer with 64 units.

### Numerical simulations

To validate DPAD in numerical simulations, we perform two sets of simulations. One set validates linear modeling to show the correctness of the four-step numerical optimization for learning. The other set validates nonlinear modeling. In the linear simulation, we randomly generate 100 linear models with various dimensionality and noise statistics, as described in our prior work^6^. Briefly, the neural and behavior dimensions are selected from 5 ≤ *n*_*y*_, *n*_*z*_ ≤ 10 randomly with uniform probability. The state dimension is selected as *n*_*x*_ = 16, of which *n*_1_ = 4 latent state dimensions are selected to drive behavior. Eigenvalues of the state transition matrix are selected randomly as complex conjugate pairs with uniform probability within the unit disk. Each element in the behavior and neural readout matrices is generated as a random Gaussian variable. State and neural observation noise covariances are generated as random positive definite matrices and scaled randomly with a number between 0.003 and 0.3 or between 0.01 and 100, respectively, to obtain a wide range of relative noises across random models. A separate random linear state-space model with four latent state dimensions is generated to produce the behavior readout noise 𝜖_*k*_, representing the behavior dynamics that are not encoded in the recorded neural activity. Finally, the behavior readout matrix is scaled to set the ratio of the signal standard deviation to noise standard deviation in each behavior dimension to a random number from 0.5 to 50. We perform model learning and evaluation with twofold cross-validation (Extended Data Fig. 1).

In the nonlinear simulations that are used to validate both DPAD and the hypothesis testing procedure it enables to find the origin of nonlinearity, we start by generating 20 random linear models (*n*_*y*_ = *n*_*z*_ = 1) either with *n*_*x*_ = *n*_*z*_ = *n*_*y*_ (Extended Data Fig. 2) or *n*_*x*_ = 2 latent states, only one of which drives behavior (Supplementary Fig. 2). We then introduce nonlinearity in one of the four model parameters (that is, *A*′, *K*, *C*_*y*_ or *C*_*z*_) by replacing that parameter with a nonlinear trigonometric function, such that roughly one period of the trigonometric function is visited by the model (while keeping the rest of the parameters linear). To do this, we first scale each latent state in the initial random linear model to find a similarity transform for it where the latent state has a 95% confidence interval range of 2*π*. We then add a sine function to the original parameter that is to be changed to nonlinear and scale the amplitude of the sine such that its output reaches roughly 0.25 of the range of the outputs from the original linear parameter. This was done to reduce the chance of generating unrealistic unstable nonlinear models that produce outputs with infinite energy, which is likely when *A*′ is nonlinear. Changing one parameter to nonlinear can change the range of the statistics of the latent states in the model; thus, we generate some simulated data from the model and redo the scaling of the nonlinearity until ratio conditions are met.

To generate data from any nonlinear model in Eq. (1), we first generate a neural noise time series *e*_*k*_ based on its covariance ∑_*e*_ in the model and initialize the state as *x*_0_ = 0. We then iteratively apply the second and first lines of Eq. (1) to get the simulated neural activity *y*_*k*_ from line 2 and then the next state ${x}_{k+1}$ from line 1, respectively. Finally, once the state time series is produced, we generate a behavior noise time series 𝜖_*k*_ based on its covariance ∑_𝜖_ in the model and apply the third line of Eq. (1) to get the simulated behavior *z*_*k*_. Similar to linear simulations, we perform the modeling and evaluation of nonlinear simulations with twofold cross-validation (Extended Data Fig. 2 and Supplementary Fig. 2).

### Neural datasets and behavioral tasks

We evaluate DPAD in five datasets with different behavioral tasks, brain regions and neural recording modalities to show the generality of our conclusions. For each dataset, all animal procedures were performed in compliance with the National Research Council Guide for Care and Use of Laboratory Animals and were approved by the Institutional Animal Care and Use Committee at the respective institution, namely New York University (datasets 1 and 2)^6,45,46^, Northwestern University (datasets 3 and 5)^47,48,54^ and University of California San Francisco (dataset 4)^21,49^.

Across all four main datasets (datasets 1 to 4), the spiking activity was binned with 10-ms nonoverlapping bins, smoothed with a Gaussian kernel with standard deviation of 50 ms (refs. ^6,14,34,103,104^) and downsampled to 50 ms to be used as the neural signal to be modeled. The behavior time series was also downsampled to a matching 50 ms before modeling. In the three datasets where LFP activity was also available, we also studied two types of features extracted from LFP. As the first LFP feature, we considered raw LFP activity itself, which was high-pass filtered above 0.5 Hz to remove the baseline, low-pass filtered below 10 Hz (that is, antialiasing) and downsampled to the behavior sampling rate of a 50-ms time step (that is, 20 Hz). Note that in the context of the motor cortex, low-pass-filtered raw LFP is also referred to as the local motor potential^50–52,105,106^ and has been used to decode behavior^6,50–53,105–107^. As the second feature, we computed the LFP log-powers^5–7,40,77,79,106,108,109^ in eight standard frequency bands (delta: 0.1–4 Hz, theta: 4–8 Hz, alpha: 8–12 Hz, low beta: 12–24 Hz, mid-beta: 24–34 Hz, high beta: 34–55 Hz, low gamma: 65–95 Hz and high gamma: 130–170 Hz) in sliding 300-ms windows at a time step of 50 ms using Welch’s method (using eight subwindows with 50% overlap)^6^. The median analyzed data length for each session across the datasets ranged from 4.6 to 9.9 min.

#### First dataset: 3D reaches to random targets

In the first dataset, the animal (named J) performed reaches to a target randomly positioned in 3D space within the reach of the animal, grasped the target and returned its hand to resting position^6,45^. Kinematic data were acquired using the Cortex software package (version 5.3) to track retroreflective markers in 3D (Motion Analysis)^6,45^. Joint angles were solved from the 3D marker data using a Rhesus macaque musculoskeletal model via the SIMM toolkit (version 4.0, MusculoGraphics)^6,45^. Angles of 27 joints in the shoulder, elbow, wrist and fingers in the active hand (right hand) were taken as the behavior signal^6,45^. Neural activity was recorded with a 137-electrode microdrive (Gray Matter Research), of which 28 electrodes were in the contralateral primary motor cortex M1. The multiunit spiking activity in these M1 electrodes was used as the neural signal. For LFP analyses, LFP features were also extracted from the same M1 electrodes. We analyzed the data from seven recording sessions.

To visualize the low-dimensional latent state trajectories for each behavioral condition (Extended Data Fig. 6), we determined the periods of reach and return movements in the data (Fig. 7a), resampled them to have similar number of time samples and averaged the latent states across those resampled trials. Given the redundancy in latent descriptions (that is, any scaling, rotation and so on on the latent states still gives an equivalent model), before averaging trials across cross-validation folds and sessions, we devised the following procedure to standardize the latent states for each fold in the case of 2D latent states (Extended Data Fig. 6). (1) We *z* score all state dimensions to have zero mean and unit variance. (2) We rotate the 2D latent states such that the average 2D state trajectory for the first condition (here, the reach epochs) starts from an angle of 0. (3) We estimate the direction of the rotation for the average 2D state trajectory of the first condition, and if it is not counterclockwise, we multiply the second state dimension by –1 to make it so. Note that in each step, the same mapping is applied to the latent states during the whole test data, regardless of condition, so this procedure does not alter the relative differences in the state trajectory across different conditions. The procedure also does not change the learned model and simply corresponds to a similarity transform that changes the basis of the model. This procedure only removes the redundancies for describing a 2D latent state-space model and standardizes the extracted latent states so that trials across different test sets can be averaged together.

#### Second dataset: saccadic eye movements

In the second dataset, the animal (named A) performed saccadic eye movements to one of eight targets on a display^6,46^. The visual stimuli in the task with saccadic eye movements were controlled via custom LabVIEW (version 9.0, National Instruments) software executed on a real-time embedded system (NI PXI-8184, National Instruments)^46^. The 2D position of the eye was tracked and taken as the behavior signal. Neural activity was recorded with a 32-electrode microdrive (Gray Matter Research) covering the prefrontal cortex^6,46^. Single-unit activity from these electrodes, ranging from 34 to 43 units across different recording sessions, was used as the neural signal. For LFP analyses, LFP features were also extracted from the same 32 electrodes. We analyzed the data from the first 7 days of recordings. We only included data from successful trials where the animal performed the task correctly by making a saccadic eye movement to the specified target. To visualize the low-dimensional latent state trajectories for each behavioral condition (Extended Data Fig. 6), we grouped the trials based on their target position. Standardization across folds before averaging was done as in the first dataset.

#### Third dataset: sequential reaches with a 2D cursor controlled with a manipulandum

In the third dataset, which was collected and made publicly available by the laboratory of L. E. Miller^47,48^, the animal (named T) controlled a cursor on a 2D screen using a manipulandum and performed a sequential reach task^47,48^. The 2D cursor position and velocity were taken as the behavior signal. Neural activity was recorded using a 100-electrode microelectrode array (Blackrock Microsystems) in the dorsal premotor cortex^47,48^. Single-unit activity, recorded from 37 to 49 units across recording sessions, was used as the neural signal. This dataset did not include any LFP recordings, so LFP features could not be considered. We analyzed the data from all three recording sessions. To visualize the low-dimensional latent state trajectories for each behavioral condition (Extended Data Fig. 6), we grouped the trials into eight different conditions based on the angle of the direction of movement (that is, end position minus starting position) during the trial, with each condition covering movement directions within a 45° (that is, 360/8) range. Standardization across folds before averaging was performed as in the first dataset.

#### Fourth dataset: virtual reality random reaches with a 2D cursor controlled with the fingertip

In the fourth dataset, which was collected and made publicly available by the laboratory of P. N. Sabes^49^, the animal (named I) controlled a cursor based on the fingertip position on a 2D surface within a 3D virtual reality environment^21,49^. The 2D cursor position and velocity were taken as the behavior signal. Neural activity was recorded with a 96-electrode microelectrode array (Blackrock Microsystems)^21,49^ covering M1. We selected a random subset of 32 of these electrodes, which had 77 to 99 single units across the recording sessions, as the neural signal. LFP features were also extracted from the same 32 electrodes. We analyzed the data for the first seven sessions for which the wideband activity was also available (sessions 20160622/01 to 20160921/01). Grouping into conditions for visualization of low-dimensional latent state trajectories (Extended Data Fig. 6) was done as in the third dataset. Standardization across folds before averaging was done as in the first dataset.

#### Fifth dataset: center-out cursor control reaching task

In the fifth dataset, which was collected and made publicly available by the laboratory of L. E. Miller^54^, the animal (named H) controlled a cursor on a 2D screen using a manipulandum and performed reaches from a center point to one of eight peripheral targets (Fig. 4i). The 2D cursor position was taken as the behavior signal. Neural activity was recorded with a 96-electrode microelectrode array (Blackrock Microsystems) covering area 2 of the somatosensory cortex^54^. Preprocessing for this dataset was done as in ref. ^36^. Specifically, the spiking activity was binned with 1-ms nonoverlapping bins and smoothed with a Gaussian kernel with a standard deviation of 40 ms (ref. ^110^), with the behavior also being sampled with the same 1-ms sampling rate. Trials were also aligned as in the same prior work^110^ with data from –100 to 500 ms around movement onset of each trial being used for modeling^36^.

### Additional details for baseline methods

For the fifth dataset, which has been analyzed in ref. ^36^ and introduces CEBRA, we used the exact same CEBRA hyperparameters as those reported in ref. ^36^ (Fig. 4i,j). For each of the other four datasets (Fig. 4a–h), when learning a CEBRA-Behavior or CEBRA-Time model for each session, fold and latent dimension, we also performed an extensive search over CEBRA hyperparameters and picked the best value with the same inner cross-validation approach as we use for the automatic selection of nonlinearities in DPAD. We considered 30 different sets of hyperparameters: 3 options for the ‘time-offset’ hyperparameter (1, 2 or 10) and 10 options for the ‘temperature’ hyperparameter (from 0.0001 to 0.01), which were designed to include all sets of hyperparameters reported for primate data in ref. ^36^. We swept the CEBRA latent dimension over the same values as DPAD, that is, powers of 2 up to 128. In all cases, we used a *k*-nearest neighbors regression to map the CEBRA-extracted latent embeddings to behavior and neural data as done in ref. ^36^ because CEBRA itself does not learn a reconstruction model^36^ (Extended Data Table 1).

It is important to note that CEBRA and DPAD have fundamentally different architectures and goals (Extended Data Table 1). CEBRA uses a small ten-sample window (when ‘model_architecture’ is ‘offset10-model’) around each datapoint to extract a latent embedding via a series of convolutions. By contrast, DPAD learns a dynamical model that recursively aggregates all past neural data to extract an embedding. Also, in contrast to CEBRA-Behavior, DPAD’s embedding includes and dissociates both behaviorally relevant neural dimensions and other neural dimensions to predict not only the behavior but also the neural data well. Finally, CEBRA does not automatically map its latent embeddings back to neural data or to behavior during learning but does so post hoc, whereas DPAD learns these mappings for all its latent states. Given these differences, several use-cases of DPAD are not targeted by CEBRA, including explicit dynamical modeling of neural–behavioral data (use-case 1), flexible nonlinearity, hypothesis testing regarding the origin of nonlinearity (use-case 4) and forecasting.

### Statistics

We used the Wilcoxon signed-rank test for all paired statistical tests.

### Reporting summary

Further information on research design is available in the Nature Portfolio Reporting Summary linked to this article.

## Online content

Any methods, additional references, Nature Portfolio reporting summaries, source data, extended data, supplementary information, acknowledgements, peer review information; details of author contributions and competing interests; and statements of data and code availability are available at 10.1038/s41593-024-01731-2.

## Supplementary information

Supplementary Information Supplementary Figs. 1–9 and Notes 1–4.

Reporting Summary

## Source data

Source Data Figs. 2–7 and Extended Data Figs. 3, 7 and 8 Statistical source data.

## Figures and Tables

**Extended Data Table 1:**
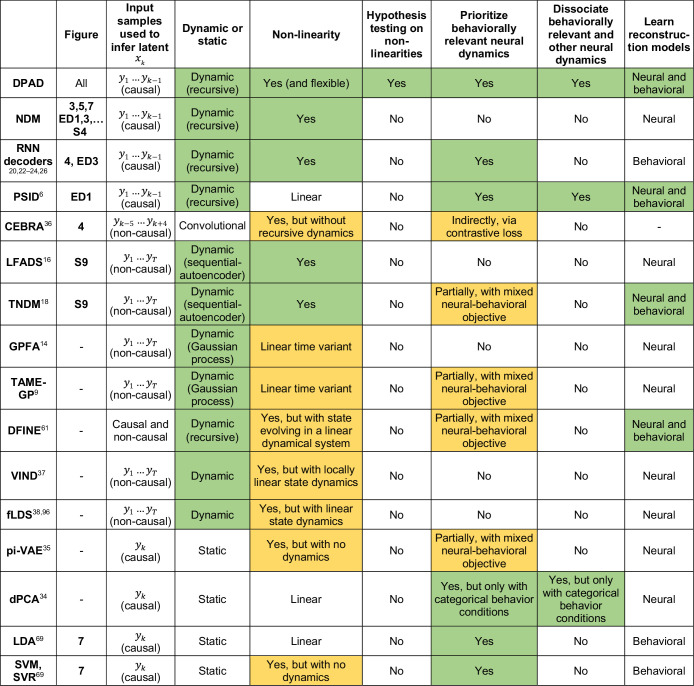
Architectural differences between DPAD and various other methods

An extended description for some columns is provided in Supplementary Note 4. Here we provide a summary. **Figure:** Figure numbers for figures that show results from the named method. ED: Extended Data Figure. S: Supplementary Figure. **Input samples used to infer latent** ***x***_***k***_**:** The subset of the input neural time series {*y*_1_, *y*_2_,…} that are used to estimate the latent variable *x*_*k*_ associated with time sample *k*. **Dynamic or static:** Dynamic models have an explicit description of the temporal structure in data, which allows them to aggregate information over time. In contrast, static models consider each given data sample on its own, and thus extract the same encoding regardless of the temporal order/structure of the input sequence. Convolutional models (for example, CEBRA) consider each small data window on its own and can’t aggregate information beyond that window, and in this sense are similar to static models. **Nonlinearity:** Nonlinear models can learn nonlinear mappings within some model elements, but unlike DPAD, they have not been flexible in terms of which model elements are made nonlinear and with what structure (note in this work we also implement NDM with flexible nonlinearity). **Hypothesis testing on nonlinearities:** DPAD is the only method that provides fine-grained control over the nonlinearity versus linearity of each model element and thus enables localization of nonlinearities and hypothesis testing regarding them (Fig. 6). **Prioritize behaviorally relevant neural dynamics**: Methods that can incorporate the reconstruction of behavior from neural data as part of their learning objective, ideally with priority. **Dissociate behaviorally relevant and other neural dynamics:** DPAD is the only dynamical nonlinear method that learns both behaviorally relevant neural dynamics and other neural dynamics, and dissociates the two into separate latent states. **Learned reconstruction models:** The reconstruction models that are natively learned by the method when extracting latents, in order to reconstruct neural or behavioral data from these learned latents.

## Data Availability

Three of the datasets used in this work are publicly available^47–49,54^. The other two datasets used to support the results are available upon reasonable request from the corresponding author. Source data are provided with this paper.

## Code availability

The code for DPAD is available at https://github.com/ShanechiLab/DPAD.
