# Supplementary material for: Dissociative and prioritized modeling of behaviorally relevant neural dynamics using recurrent neural networks
Source: Nat Neurosci. 2024 Sep 6;27(10):2033–45. doi: 10.1038/s41593-024-01731-2 (PMC11452342; doi:10.1038/s41593-024-01731-2)
Supplement: Supplementary file 1 — Supplementary Figs. 1–9 and Notes 1–4. [file 41593_2024_1731_MOESM1_ESM.pdf]

nature neuroscience

Supplementary information

https://doi.org/10.1038/s41593-024-01731-2

Dissociative and prioritized modeling of behaviorally relevant neural dynamics using recurrent neural networks

In the format provided by the authors and unedited

Supplementary Information

Contents

- Supplementary Figures: **Supplementary Figs. 1-9**
- Supplementary Notes: **Supplementary Notes 1-4**

Note: please refer to the main text and **Methods** for equations (1)-(14).

Supplementary Figures

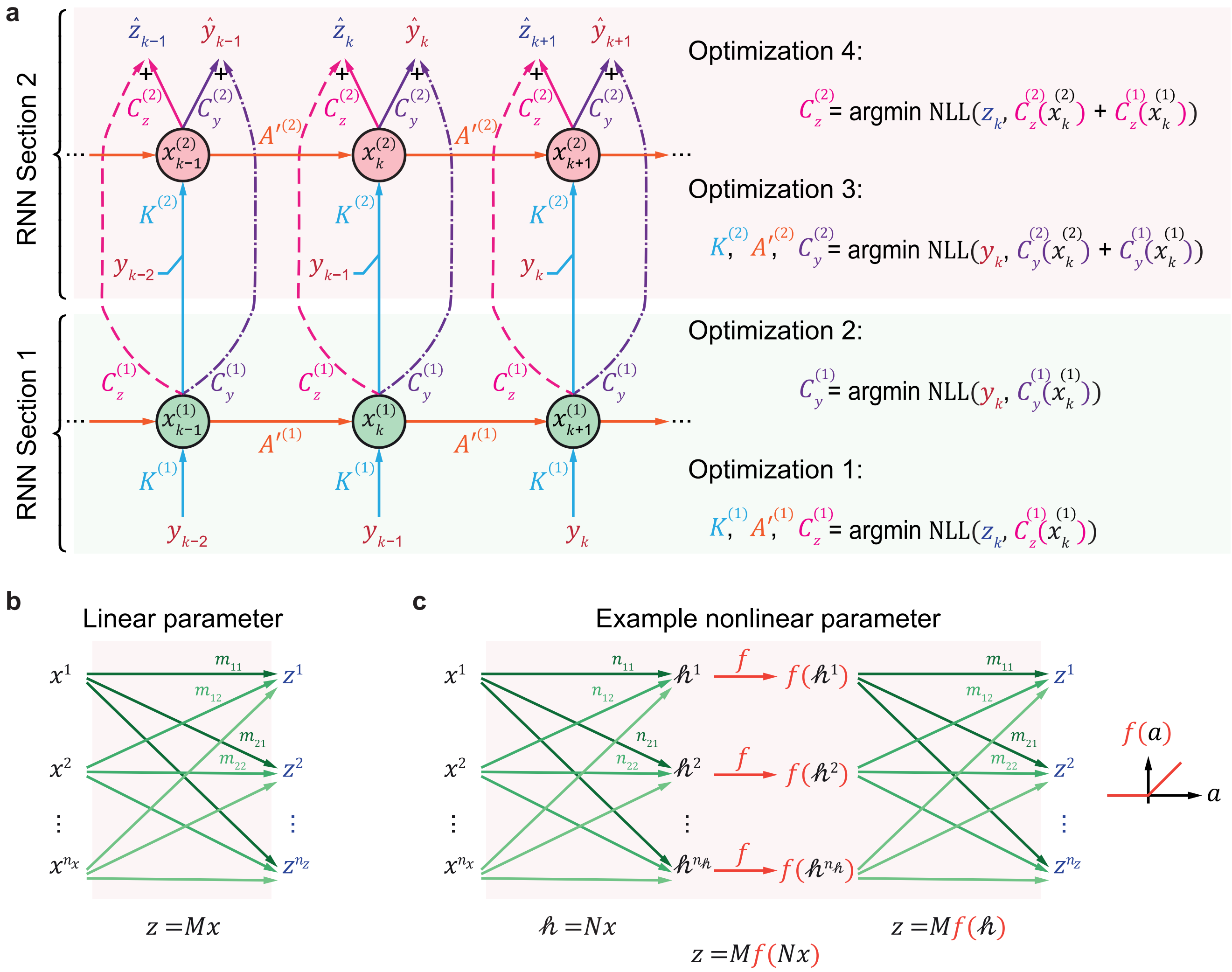

Supplementary Fig. 1 | Detailed computation graph for the two-section RNN model in DPAD and all the four connected optimization steps for learning the model parameters.

a, The computation graph is shown as unfolded[111] in time to make the relation between the time indices of the latent states, neural data, and neural and behavioral predictions clearer. Learning consists of 4 connected numerical optimization problems (**Methods**): 1) Learn $A'^{(1)}$, $K^{(1)}$, and $C_z^{(1)}$ by fitting an RNN that minimizes the negative log-likelihood (NLL) of behavior given the RNN states $x_k^{(1)}$; 2) Learn $C_y^{(1)}$ by fitting a feed-forward neural network that minimizes the NLL of neural activity given the extracted $x_k^{(1)}$; 3) Learn $A'^{(2)}$, $K^{(2)}$, and $C_y^{(2)}$ by fitting an RNN that as input takes neural activity and the states extracted from the first optimization step, i.e., $x_k^{(1)}$; this RNN minimizes the aggregate NLL of neural activity given its states $x_k^{(2)}$ and the first set of states $x_k^{(1)}$ (note that $x_k^{(1)}$ is computed in optimization step 1 and has known values when solving subsequent optimization steps); and 4) Learn $C_z^{(2)}$ by fitting a feed-forward neural network that minimizes the aggregate NLL of behavior given the states extracted in optimization step 3, i.e., $x_k^{(2)}$. Step 4 has the option of alternatively learning a more general aggregate C_z that takes both sets of states as input and replaces both $C_z^{(1)}$ and $C_z^{(2)}$, to support non-additive combination of the latent states in predicting behavior (**Methods**). The first two optimization steps learn to extract the behaviorally relevant latent states $x_k^{(1)}$ from past neural activity and learn their mapping to future neural-behavioral data. The last two optimization steps, which are optional, learn to extract additional latent states $x_k^{(2)}$ from past neural activity and learn

their mapping to any residual future neural-behavioral dynamics not already predicted by $x_k^{(1)}$. The dimension of $x_k^{(1)}$ and $x_k^{(2)}$ are hyperparameters that need to be determined by the user. If only learning of behaviorally relevant neural dynamics is of interest, the dimension of $x_k^{(2)}$ can be set to zero, removing the second section of the RNN and the need for optimization steps 3-4. Conversely, if prioritized learning of neural dynamics relevant to behavior is not of interest (i.e., as in NDM), the dimension of $x_k^{(1)}$ can be set to zero, removing the first section of the RNN and the need for optimization steps 1-2. The architecture of the feed-forward neural network that constructs each model parameter (see **b**,**c**) can be determined by the user as part of the "nonlinearity setting" as illustrated in **Fig. 1c**. Manual specification of nonlinearity settings is useful for example for using linear DPAD and for hypothesis testing with individual nonlinearities in **Fig. 6**. Alternatively, as illustrated in **Fig. 1d,** the "nonlinearity setting" can be automatically selected among a range of architectures using an inner-cross-validation within the training data, which we refer to as flexible nonlinearity (e.g., **Fig. 3 and 5**). For Gaussian-distributed data, assuming isotropic residuals (**Methods**), the NLL in the optimization objectives is proportional to the sum of squared errors. For non-Gaussian data distributions (e.g., categorical behavior), in addition to using the appropriate (e.g., categorical) NLL to form the optimization objective, we also change the associated readout architecture to form that NLL. For example, we add one output per class and a Softmax normalization to $C_z^{(1)}$ and $C_z^{(2)}$ when behavior z_k has a categorical distribution (**Methods**). Moreover, in the non-Gaussian case, NLLs from steps 1-2 are again incorporated as preexisting NLLs into optimization steps 3-4 so that the latter optimization steps complement the predictions from steps 1-2 (**Methods**). **b**,**c**, Any one or all of the model parameters from **a** can be either a linear matrix (**b**), or in general an arbitrary multilayer feed-forward neural network (**c**). The example feed-forward network in **c** has one hidden layer with h units and uses a rectified linear unit (ReLU) activation function for the hidden layer. The recursion parameter A' can also implement an LSTM recursion as one option for nonlinearity (**Methods**).

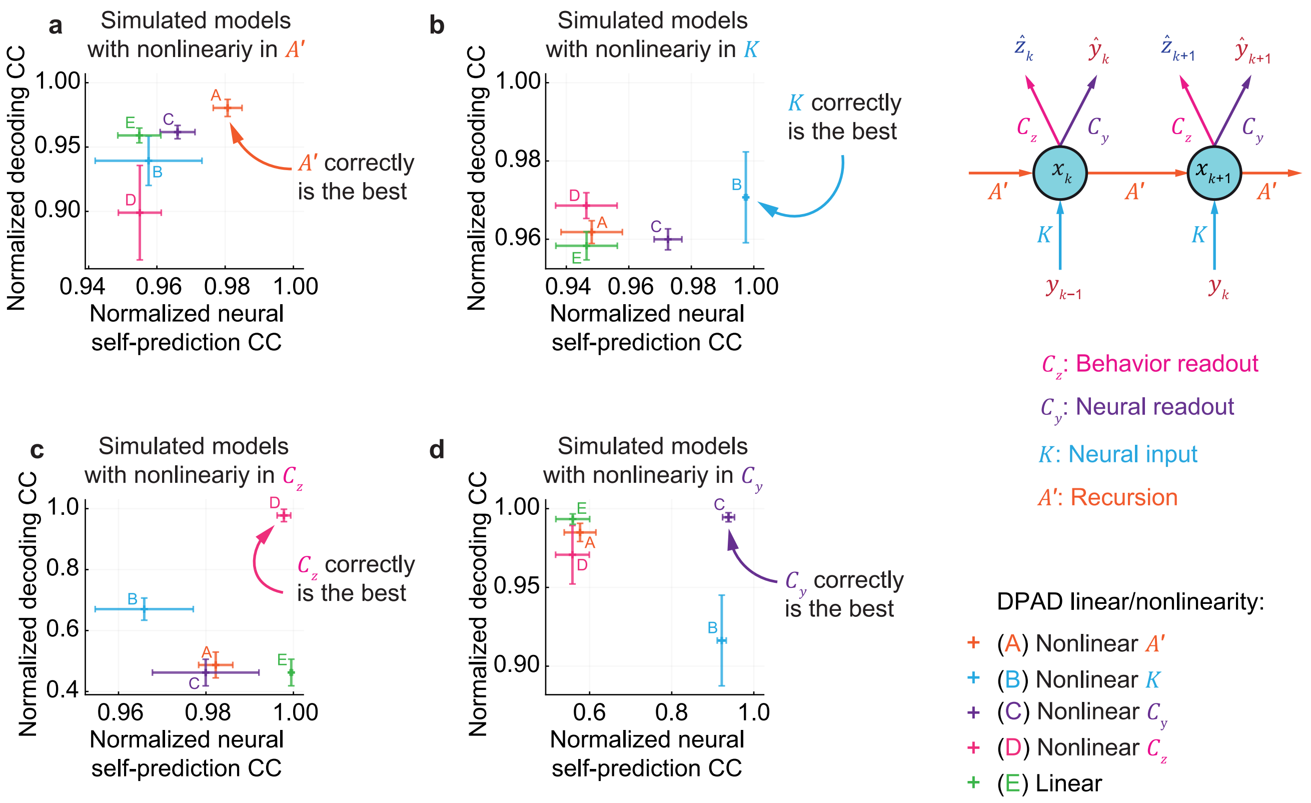

Supplementary Fig. 2 | DPAD's localization of nonlinearities is accurate in numerical simulations.

a-d, Same as **Extended Data Fig. 2** (with pluses and whiskers defined in the same way), showing accurate localization of nonlinearities in a different set of 20 simulated systems with both behaviorally relevant and irrelevant latent states such that all optimization steps are used in the modeling (N = 20 random models in all panels) (**Methods**). Similar to **Extended Data Fig. 2**, the multi-step optimization learning approach in DPAD accurately localizes the nonlinearities regardless of their true location in these simulations.

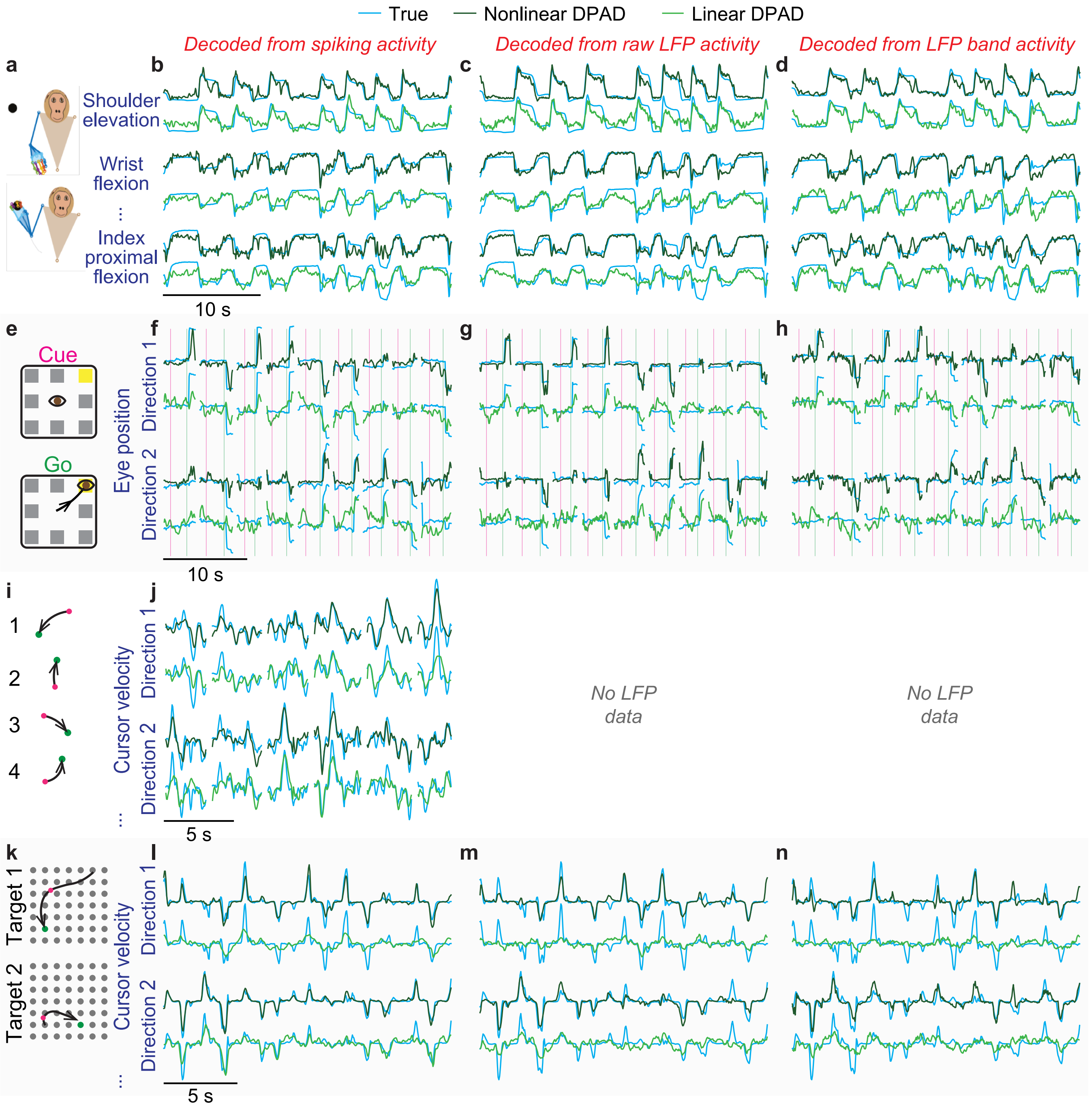

Supplementary Fig. 3 | Example behavioral trajectories and nonlinear versus linear decoding using each neural modality.

a, The 3D reach task. **b**, Example true and predicted behavior trajectories in the test data for multiple joints, using nonlinear and linear DPAD models from **Fig. 2**. For behavior decoding statistics across all data see **Fig. 2**. **c**, Same as **b** for behavior decoding using raw LFP activity. **d**, Same as **b** for behavior decoding using LFP band power activity. **e**-**h**, Same as **a**-**d** for the second dataset, with saccadic eye movements. **i**-**j**, Same as **a**-**d** for the third dataset, with sequential cursor reaches controlled via a 2D manipulandum. **k**-**n**, Same as **a**-**d** for the fourth dataset, with random grid virtual reality cursor reaches controlled via fingertip position.

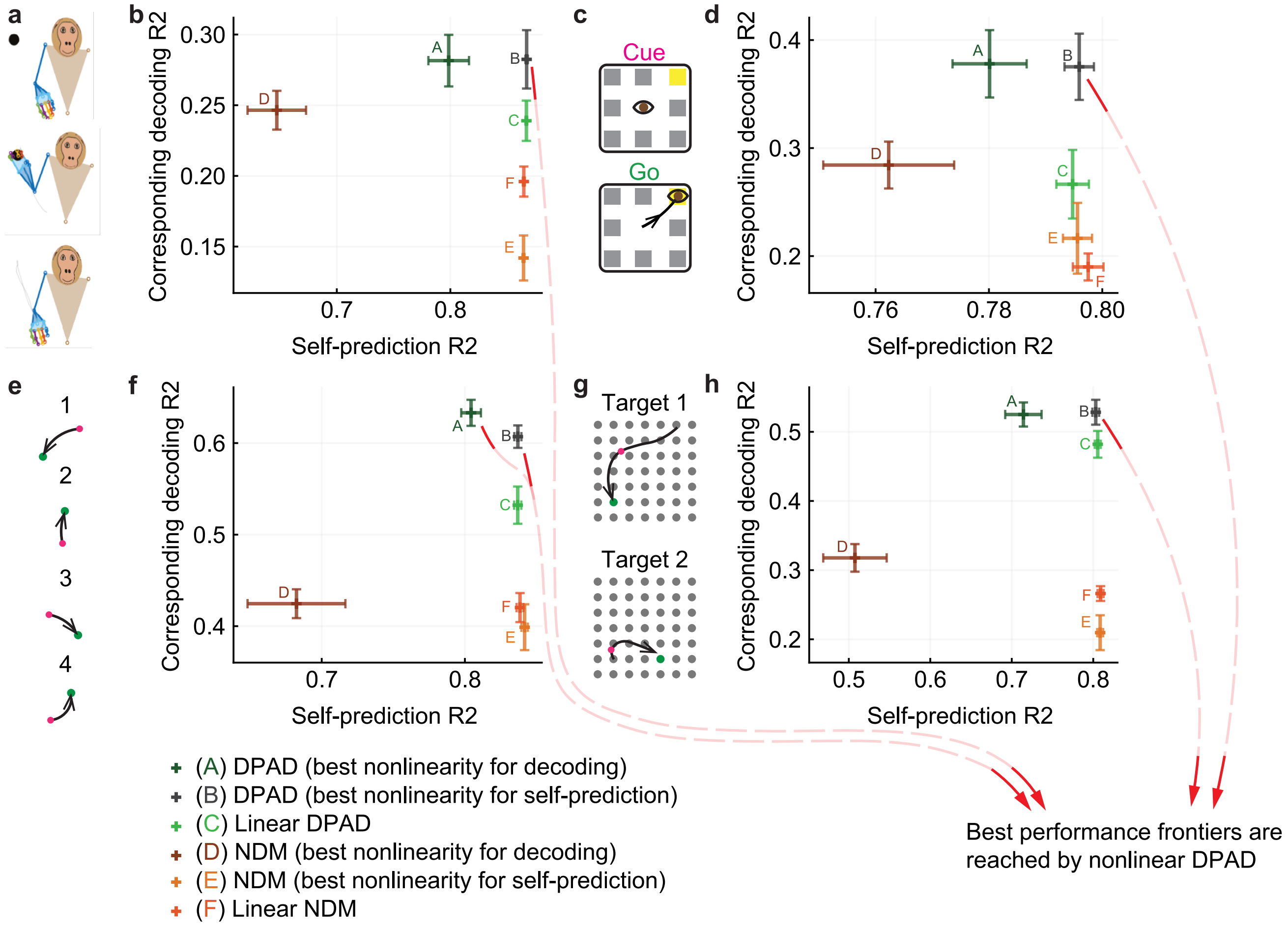

Supplementary Fig. 4 | DPAD remains on the best performance frontier for predicting neural-behavioral dynamics, even when performances are evaluated with coefficient of determination (R2) instead of correlation coefficient (CC).

a-h, Figure content is parallel to **Fig. 3** (with pluses and whiskers defined in the same way), but shows the R2 for performances for the same models instead of showing their CC (*N* = 35 session-folds in **b**,**d**,**h** and *N* = 15 session-folds in **f**).

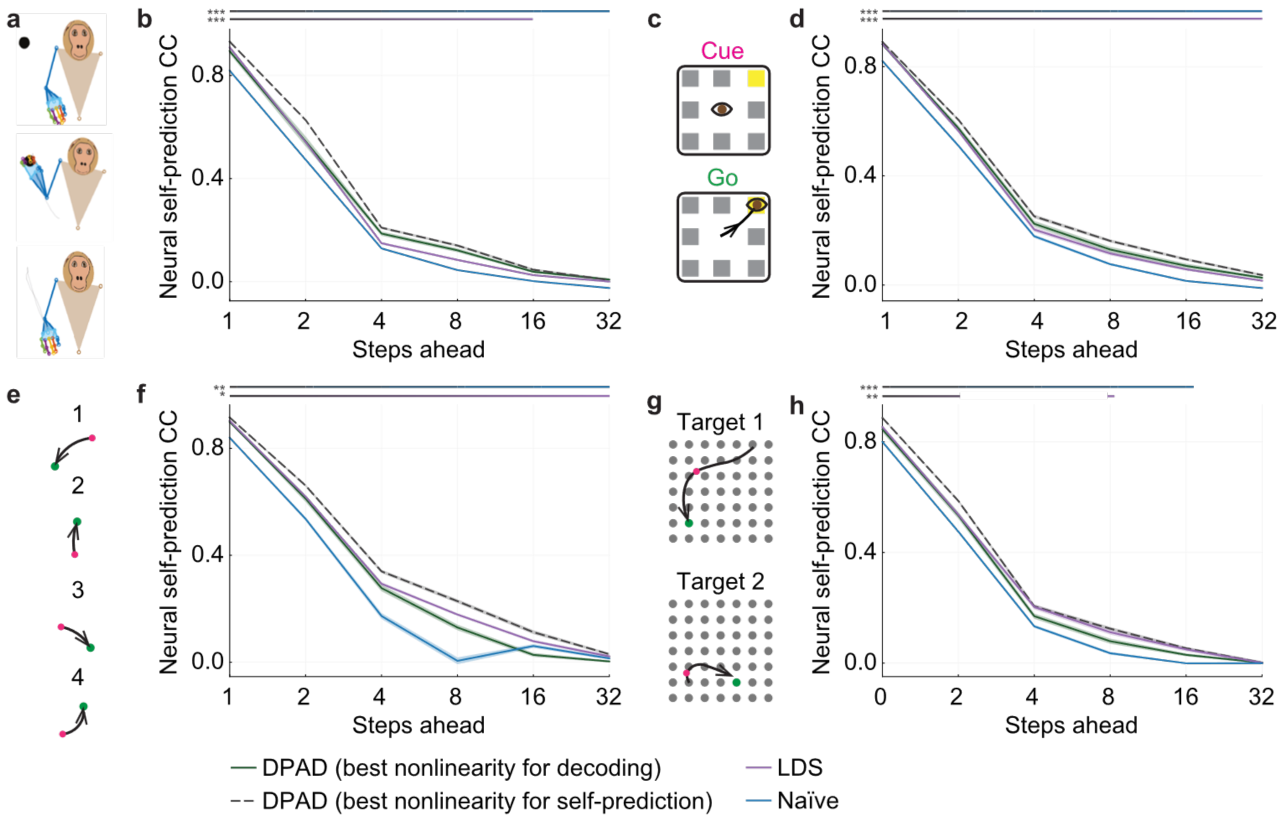

Supplementary Fig. 5 | DPAD can also be used for multi-step-ahead forecasting of neural data.

a-h, Similar to **Extended Data Fig. 4**, but showing the multi-step-ahead neural self-prediction accuracy for DPAD and its comparison with that of a baseline Naïve predictor, which predicts future neural activity as the current activity. A Naïve predictor represents the self-prediction performance expected simply due to the autocorrelations and smoothness of neural data even without a model. Solid lines and shaded areas are defined as in **Fig. 5b** (*N* = 35 session-folds in **b**,**d**,**h** and *N* = 15 session-folds in **f**). Importantly, these results are again obtained without any retraining or finetuning, with m-step-ahead forecasting done by repeatedly ($m-1$ times) passing the neural predictions of the model as its neural observation in the next time step (**Methods**). Across the number of steps ahead, the statistical significance of a one-sided pairwise comparison between nonlinear DPAD vs Naïve is shown with the blue top horizontal line with p-value indicated by asterisks next to the line as defined in **Fig. 2b**. Similar pairwise comparison between nonlinear DPAD vs LDS modeling is shown with the purple top horizontal line. DPAD consistently achieved significantly higher self-prediction accuracy than a Naïve predictor, suggesting that it is learning temporal dynamics beyond simply the smoothness in data.

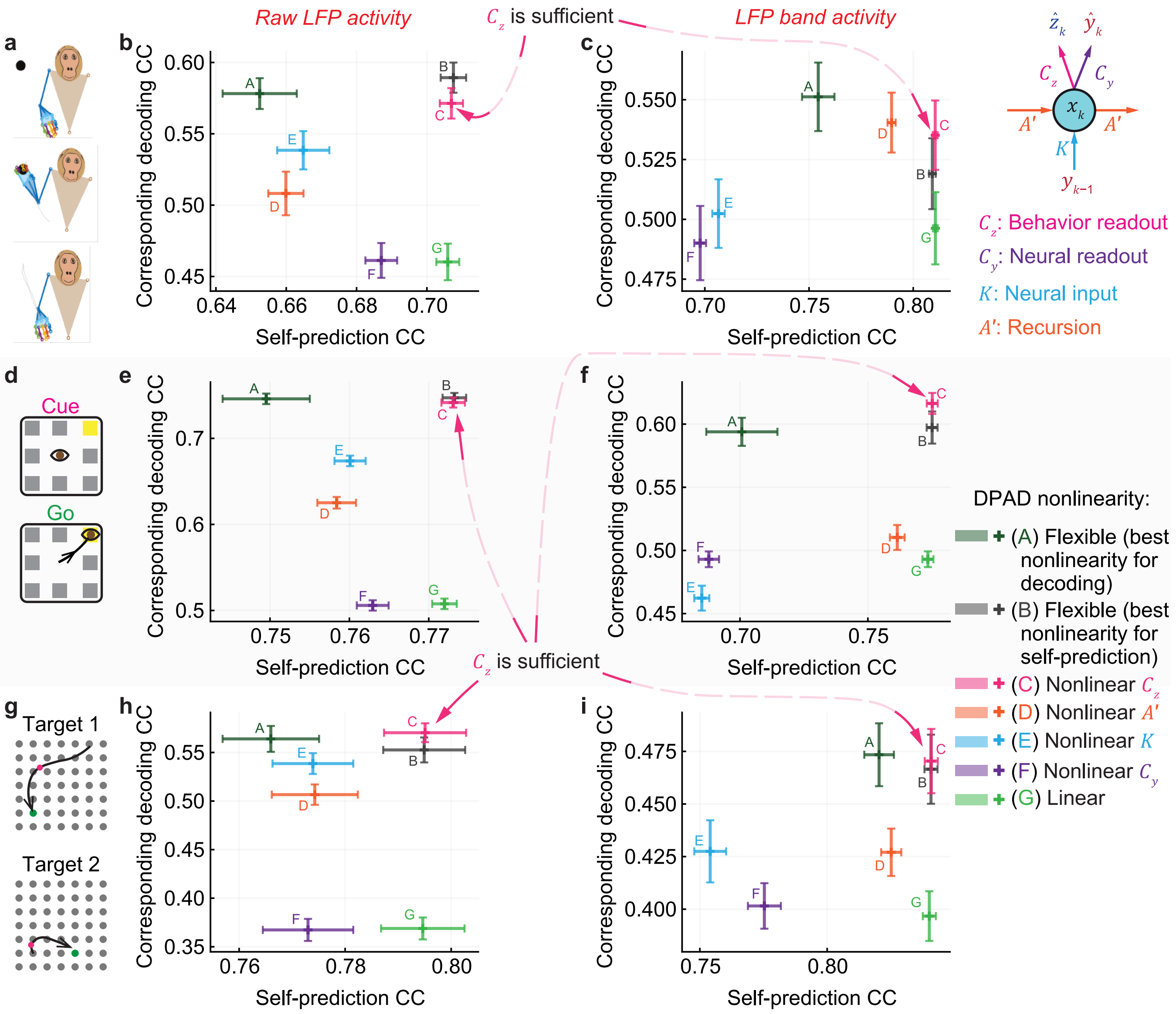

Supplementary Fig. 6 | In hypothesis testing for raw LFP and LFP band power activity, DPAD again provides evidence that nonlinearities can be largely captured by the behavior readout of the model across datasets. **a-i**, Figure content is parallel to **Fig. 6** (with pluses and whiskers defined in the same way), but now shown for raw LFP (**b**,**e**,**h**) and LFP band power (**d**,**f**,**i**) activity (N = 35 session-folds in all panels). The behavior readout nonlinearity does better than every other individual nonlinearity and comparable to when nonlinearity is flexibly chosen to be in all or any combination of parameters. As in **Fig. 6**, hypothesis testing is done by considering both behavior decoding (vertical axis) and neural self-prediction (horizontal axis), that is, in terms of being on the best performance frontier (**Methods**). This result provides evidence that nonlinear behavior readout is largely sufficient for predicting neural-behavioral data from past neural activity for both LFP modalities across all datasets.

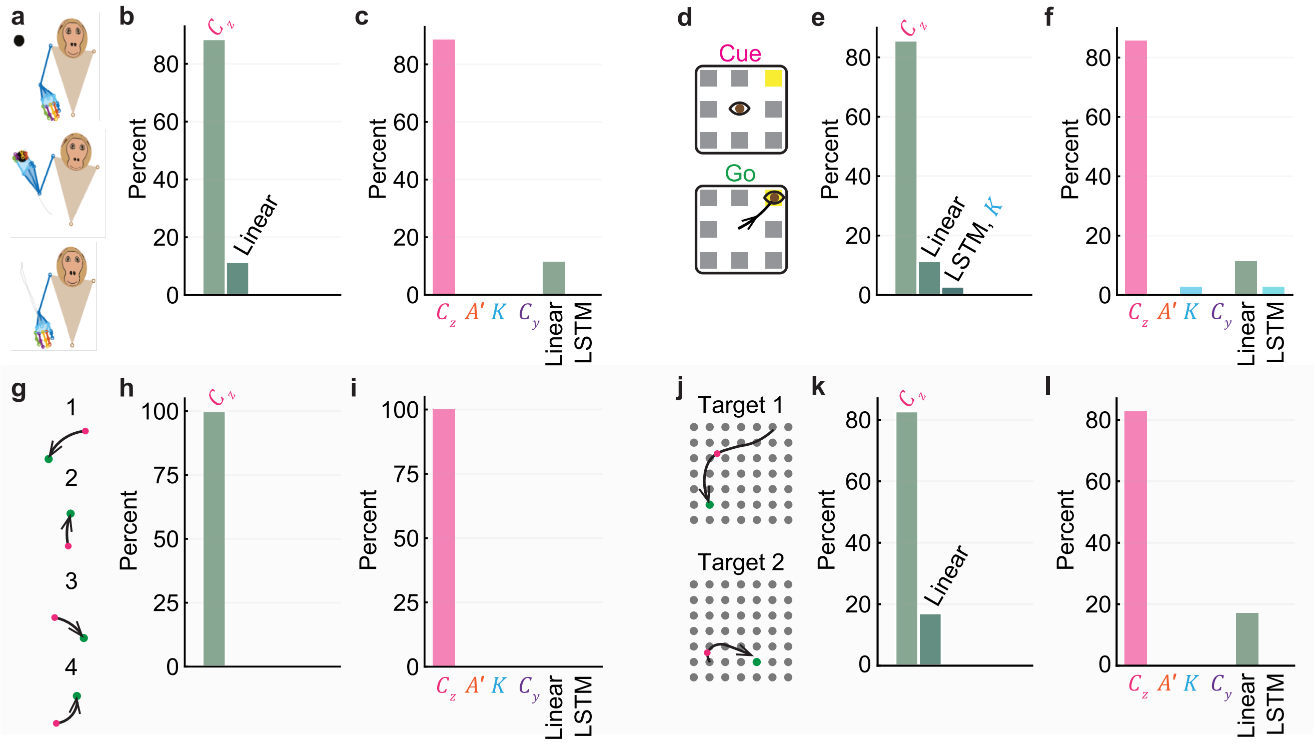

Supplementary Fig. 7 | DPAD with its flexible nonlinearity automatically finds an appropriate subset of parameters to make nonlinear to predict the training data well — but this procedure is not sufficient for localization, in which finding a minimal subset of parameters that can summarize all nonlinearities is of interest (Fig. 6).

a, The 3D reach task. **b**, The percentage of session-folds (out of *N* = 35 session-folds) that had a specific parameter set automatically selected as nonlinear, when using DPAD with flexible selection of nonlinearity based on self-prediction. The cross-validated performances for the same selected models are shown in **Fig. 3**. The label of each bar indicates the nonlinear set. LSTM indicates that an LSTM was used for recurrence (replaces A', **Methods**). As is the case here, in DPAD, unless otherwise noted, we use a flexible nonlinearity whereby we build models with nonlinearities in different parameters and select one model among them that achieves the best neural self-prediction (or decoding) in the training data (**Methods**). We note, however, that this operation is not sufficient for localizing the nonlinearity (**Fig. 6**) because the inclusion of a parameter as part of the nonlinearity does not indicate whether or not nonlinearity in that parameter is indispensable; instead, as long as the nonlinearity of a parameter does not hurt the criteria, it may be set to be nonlinear even if its nonlinearity is not necessary. Thus, to localize the nonlinearity, we explore whether nonlinearities can be isolated in individual parameters in **Fig. 6**. Nevertheless, here we show the statistics for automatic selection of nonlinearities just to show their interesting consistency with our localization results. **c**, The data in **b** shown in a different way to demonstrate i) what percentage of final flexible models had nonlinearity in each model parameter regardless of whether other parameters were linear or nonlinear, ii) what percentage used an LSTM, and iii) what percentage was fully linear. **d-f**, Same as **a-c** for the second dataset, with saccadic eye movements (*N* = 35 session-folds). **g-i**, Same as **a-c** for the third dataset, with sequential cursor reaches controlled via a 2D manipulandum (*N* = 15 session-folds). **j-l**, Same as **a-c** for the fourth dataset, with random grid virtual reality cursor reaches controlled via fingertip position (*N* = 35 session-folds). The first 16 latent state dimensions in DPAD are learned using the first two optimization steps and the remaining dimensions are learned using the last two optimization steps (i.e., n_1 = 16).

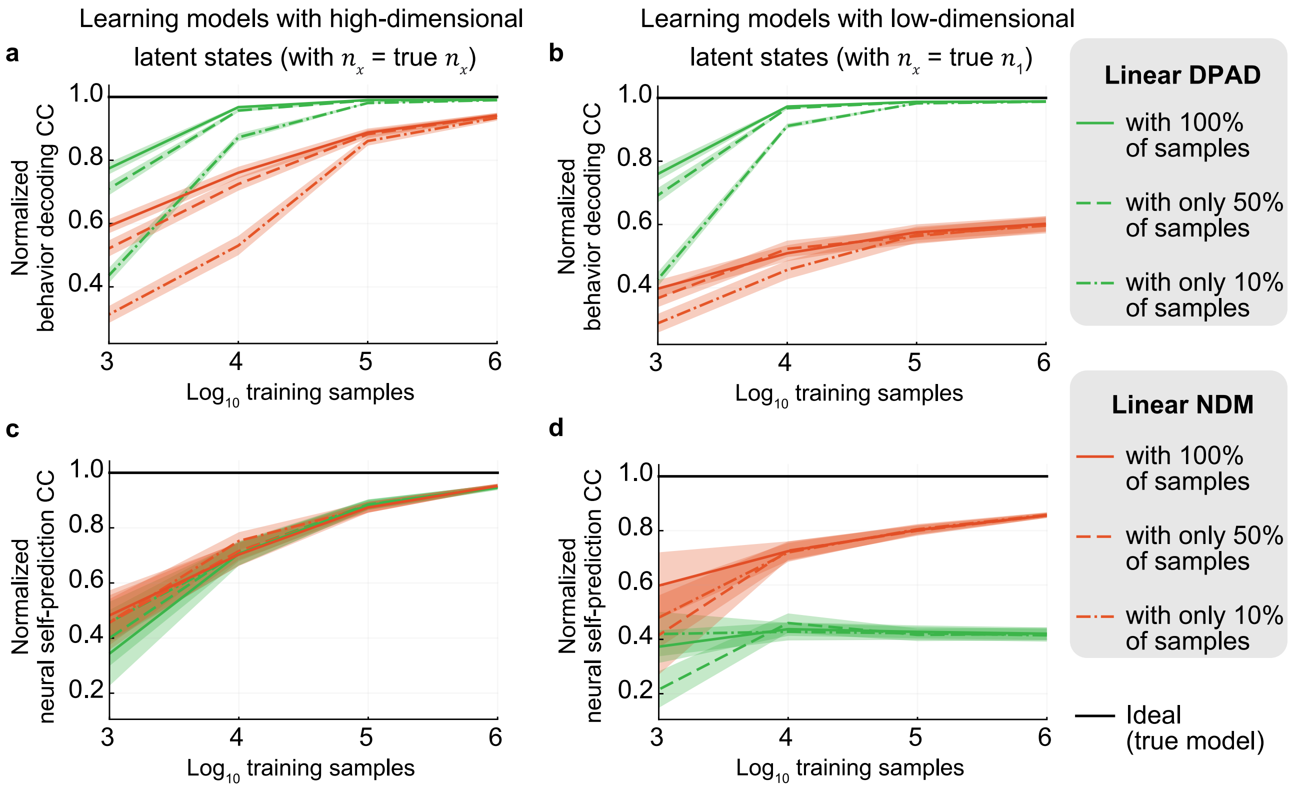

Supplementary Fig. 8 | DPAD accurately learns behavioral and neural dynamics in numerical simulations even if behavioral samples are intermittently available in the training data.

a-d, Figure content is parallel to **Extended Data Fig. 1** but shows the prediction performance for linear DPAD and linear NDM models that were trained with an intermittently sampled version of the behavior time series. To generate the training data in each case, the behavior time series was subsampled by keeping each sample based on a random draw, for example to keep 10% of samples for training, each time sample of behavior data was kept with a 10% probability. The other unkept behavior samples in the training data were treated as unmeasured data, emulating a scenario where behavior is only intermittently sampled. In all panels, solid lines and shaded areas are defined as in **Fig. 5b** (N = 100 random models). Even when as little as 10% of behavior samples are "measured" during the training, linear DPAD reaches toward ideal behavior and neural prediction performance (the performance achieved by the true model). **a** and **b** show the normalized behavior decoding for high and low latent state dimensions, respectively. **c** and **d** show the normalized neural self-prediction for high and low latent state dimensions, respectively. As in **Extended Data Fig. 1**, **b** shows that DPAD reaches ideal behavior prediction even with low-dimensional latent states, due to its prioritized learning of behaviorally relevant neural dynamics, as opposed to aiming to explain the most neural variance. Moreover, as in **Extended Data Fig. 1**, **c** shows that given enough latent state dimensions (same as the true model), using its last two optimization steps (**Supplementary Fig. 1a**), DPAD learns to predict the overall neural dynamics as accurately as NDM, converging toward ideal neural self-prediction at the same pace.

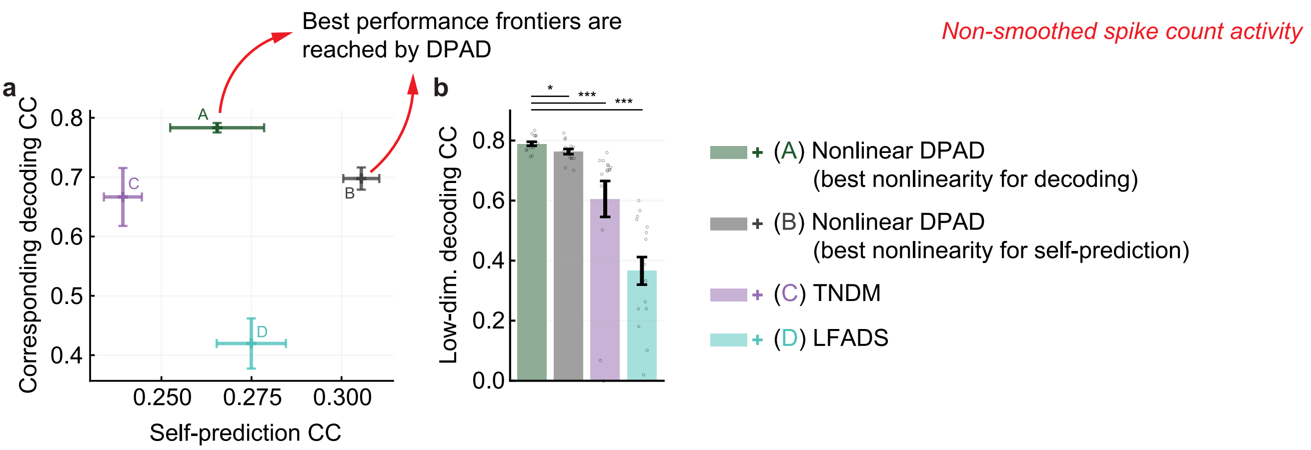

Supplementary Fig. 9 | DPAD can also model non-smoothed spike counts.

a, Result of modeling non-smoothed spike counts and behavior in the third dataset, with sequential cursor reaches controlled via a 2D manipulandum. Cross-validated neural self-prediction accuracy achieved by each method shown on the horizontal axis versus the corresponding behavior decoding accuracy on the vertical axis. Latent state dimension for each method in each session and fold is chosen (among powers of 2 up to 128) as the smallest that reaches peak neural self-prediction in training data or reaches peak decoding in training data, whichever is larger (**Methods**). Pluses and whiskers are defined as in **Fig. 3** (N = 15 session-folds). Note that the behavior decoding and neural self-prediction in DPAD are causal and only use past neural data. For comparison, we also show results for two non-causal sequential auto-encoder methods: 1) LFADS[16], and 2) a subsequent work related to LFADS termed Targeted Neural Dynamical Modeling (TNDM)[18], which was concurrently developed with our work[44,56] and models non-smoothed spike count data along with a continuous behavior. We used the python library[‡] published alongside ref. 18 to run both methods. We sweep the total LFADS/TNDM factor dimension in the same range as the total state dimension for DPAD (among powers of 2 up to 128). For all DPAD variations, the first 16 latent state dimensions are learned using the first two optimization steps to be behaviorally relevant, and the remaining dimensions are learned using the last two optimization steps (i.e., n_1 = 16). Similarly, for TNDM, the factors for decoding behavior are set to be 16-dimensional. To allow for the best possible behavior decoding from these factor dimensions in TNDM, we use the version of TNDM that fits a different dedicated non-causal regression for each different time point during a trial: this non-causal regression goes from the factors during the whole trial to predict the behavior at each time point during the trial. Finally, given that LFADS and TNDM can only process fixed-length data, we pass the data to LFADS/TNDM in non-overlapping 1s segments as done previously for similar sequential variational autoencoders[95]. **b**, Cross-validated decoding accuracy of each method at the dimension for which DPAD (with best nonlinearity for decoding) reaches within 5% of its peak decoding accuracy in training data across all latent state dimensions. Bars, whiskers, dots, and asterisks are defined as in **Fig. 2b** (N = 15 session-folds). DPAD achieves more accurate decoding than LFADS/TNDM using low-dimensional latent states. These results highlight that the strength of DPAD for prioritized and nonlinear dynamical dimensionality reduction (use-case 3) and for modeling the overall neural-behavioral data (use-case 1) extend to non-smoothed spike counts. Note that TNDM does not achieve multiple use-cases of DPAD including dynamic transformation of raw LFP (use-case 2), hypothesis testing regarding the origin of nonlinearity (use-case 4), and application to categorical or intermittent behaviors (use-case 5), so cannot be compared in these use-cases.

[‡] https://github.com/HennigLab/tndm

Supplementary Notes

Supplementary Note 1: Predictor form of dynamical system models

The model used by DPAD is provided in equation (1). DPAD is not an extension of any existing linear modeling method. However, DPAD's model formulation supports linear models as a special case by setting all parameters to be linear (**Methods**). Note that even in the special linear case, DPAD learns the model based on numerical optimization methods rooted in deep learning and thus is fully distinct from prior linear modeling methods such as PSID[6] as expanded on in the main text.

Here, we expand on the motivation and intuition behind DPAD's RNN model formulation, which is in predictor form. To make understanding the predictor form simpler, we first present the predictor form of a linear dynamical system. As a general linear dynamical system, neural activity $y_k \in \mathbb{R}^{n_y}$ and behavior $z_k \in \mathbb{R}^{n_z}$ can be jointly modeled as

$$\begin{cases} x'_{k+1} = A\ x'_k + w'_k \\ y_k = C_y\, x'_k + v'_k \\ z_k = C_z\, x'_k + \epsilon'_k \end{cases} \tag{15}$$

where $x'_k \in \mathbb{R}^{n_x}$ is a latent variable, w'_k and v'_k are Gaussian white noises, and ϵ'_k is a general random process that is independent of neural activity and represents any behavior dynamics that are not encoded in neural activity[6]. Given the above linear model, the latent states can be estimated from the neural activity y_k using a Kalman filter

$$\hat{x}_{k+1|k} = A\, \hat{x}_{k|k-1} + K\left(y_k - C_y\, \hat{x}_{k|k-1}\right) \tag{16}$$

where K is the steady-state Kalman gain[67,68] and $\hat{x}_{k+1|k}$ is the predicted state at time $k+1$ based on past neural activity up to time k. Equation (15) can be equivalently[67,68] written in terms of the steady-state Kalman estimated states $\hat{x}_{k|k-1}$ as

$$\begin{cases} x_{k+1} = A\ x_k + K e_k \\ y_k = C_y\, x_k + e_k \\ z_k = C_z\, x_k + \epsilon_k \end{cases} \tag{17}$$

where $x_k \triangleq \hat{x}_{k|k-1}$ and e_k and ϵ_k are respectively the parts of neural and behavior signals that cannot be predicted from past neural activity (i.e., $\{y_{k'} \in \mathbb{R}^{n_y}: 0 \le k' < k\}$). Equivalently, by replacing e_k from the first line with its value from the second line, we can also write equation (17) as

$$\begin{cases} x_{k+1} = A' \, x_k + K y_k \\ y_k = C_y \, x_k + e_k \\ z_k = C_z \, x_k + \epsilon_k \end{cases} \tag{18}$$

where $A' \triangleq A - KC_y$. Equations (15) and (18) are different latent descriptions that realize the same second order statistics for the stationary observation time series y_k and z_k, and thus are equivalent[67,68]. In the terminology of ref. 68, equations (15) and (18) are different internal descriptions that have the same external description, that is, their differences are only in properties that are not measurable and are thus not distinguishable (e.g., the exact value of the latent state). These two formulations are referred to as the stochastic and predictor forms, respectively[67,68].

Note that since equation (18) is based on the steady-state Kalman filter, it would give different values for the latent state compared with a non-steady-state Kalman filter (with a non-steady-state gain). When the Kalman filter converges to steady-state, the aforementioned latent state difference would be minimal (for all data points except for those prior to convergence, which are points very close to the beginning of a given time series). Also, note that equations (15) and (18) have the same second order statistics in their observations y_k and z_k as explained in textbook refs. 67,68.

Finally, note that while the first line of the predictor form formulation in equation (18) does not show an explicit noise term in the latent dynamics, these latent dynamics are written as a function of the neural observation of the previous time step (i.e., y_k), which itself is stochastic and has added noise e_k as can be seen in second line of equation (18). In fact, as noted earlier, stochastic and predictor form formulations of equations (15) and (18) are equivalent[67,68]. However, the stochastic form (equation (15)) has redundancy, meaning that there are infinitely many formulations (even beyond similarity transforms, defined in equation (21)) for the same linear dynamical system (see Faurre's theorem in ref. 67). In contrast, the predictor form (equation (18)) for a given observation statistic is unique (within a similarity transform)[6,67,68]. Moreover, the state equation in the predictor form directly describes how information from observations y_k should be combined with the current states (x_k) to get the states in the next time step, i.e., as in the first line of equation (18). While for linear models the associated predictor form can be directly computed from the stochastic form (based on the Kalman filter as we did here to derive equation (18)), this is not possible for nonlinear models in general. As such, it can be desirable to directly learn nonlinear models in predictor form, such that the learned models can be directly used for prediction of latent states from observations. As

we show here, this predictor form learning can be done since the predictor form can be thought of as an RNN, and thus can be learned from data using numerical optimization tools that are commonly used in deep learning.

To see how DPAD's RNN-based model formulates a dynamical system in predictor form – albeit a nonlinear predictor form – we can look at equation (18). We can then replace each multiplication between a model parameter and a vector (e.g., $A'x_k$) in equation (18) with a multi-input-multi-output function applied to an input vector (e.g., function $A'(\cdot)$, applied to x_k). Rewriting all matrix multiplications as multi-input-multi-output functions we get

$$\begin{cases} x_{k+1} = A'(x_k) + K(y_k) \\ y_k = C_y(x_k) + e_k \\ z_k = C_z(x_k) + \epsilon_k \end{cases} \tag{19}$$

where each function (e.g., $A'(\cdot)$) is a parameter to be learned. We can now allow any subset of the parameters in this model to be general nonlinear functions, implemented in the form of arbitrary multilayer neural networks. This gives the predictor model form for the DPAD model in equation (1), which captures nonlinearity unlike linear dynamical models. Also, DPAD learns these nonlinear parameters through the multi-step learning approach that we developed, which in each step leverages the numerical optimization tools commonly used in deep learning (**Supplementary Fig. 1a**, **Supplementary Note 2**, **Methods**); this is distinct from analytical methods used in linear modeling[6].

Given that the DPAD model in equation (1) is constructed in the predictor form, even when parameters are nonlinear, the model can still be readily used to estimate the latent states x_k given the neural observations y_k, and to decode behavior z_k. To do this, we run the first line of equation (1) to estimate the latent state and then pass this state through the learned C_y or C_z functions in the second or the third line to predict neural activity or decode behavior, respectively. All this can be done causally, only using past neural activity.

Supplementary Note 2: The four-step optimization formulation for learning in DPAD

In this note, we formulate the DPAD model such that the behaviorally relevant latent states are dissociated from the other states as shown in equation (2), which allows the model to be learned with the four-step numerical optimization approach outlined in **Methods** (**Supplementary Fig. 1a**). The behaviorally relevant latent states, denoted by $x_k^{(1)} \in \mathbb{R}^{n_1}$, are defined as those that influence behavior. Thus, observing

behavior would be informative of such behaviorally relevant latent states, a concept that is known as observability[68].

To make the exposition simpler, here, we start by showing how the special case of a fully linear dynamical system can be written in the form of equation (2) with the model parameters taken to be the special case of linear matrix multiplications (see equation (24) below). Subsequently, we make the functions in equation (2) general nonlinear neural networks, and by doing so we enable nonlinear modeling in DPAD (**Methods**).

For the special case of a linear dynamical system as in equation (18), we can compute the number of behaviorally relevant latent state dimensions n_1 based on the concept of observability, as the rank of the observability matrix associated with matrices (A', C_z)[6]. Based on this concept, we can also dissociate the parts of the latent state x_k that are observable versus unobservable through behavior (without loss of generality) as

$$\begin{cases} \begin{bmatrix} x_{k+1}^{(1)} \\ x_{k+1}^{(2)} \end{bmatrix} = \begin{bmatrix} A'_{11} & 0 \\ A'_{21} & A'_{22} \end{bmatrix} \begin{bmatrix} x_k^{(1)} \\ x_k^{(2)} \end{bmatrix} + \begin{bmatrix} K^{(1)} y_k \\ K'^{(2)} y_k \end{bmatrix} \\ \quad y_k = C_y^{(1)} x_k^{(1)} + C_y^{(2)} x_k^{(2)} + e_k \\ \quad z_k = C_z^{(1)} x_k^{(1)} + \epsilon_k \end{cases} . \tag{20}$$

by applying theorem 3.8 from ref. 68 to the first and third lines of equation (18). More specifically, the mentioned theorem states that a nonsingular square matrix E exists that would give the latent states and the model parameters of equation (20) from those of equation (18) via the following transformation

$$\begin{bmatrix} x_k^{(1)} \\ x_k^{(2)} \end{bmatrix} = E x_k, \quad \begin{bmatrix} A'_{11} & 0 \\ A'_{21} & A'_{22} \end{bmatrix} = E A' E^{-1}, \quad \begin{bmatrix} K^{(1)} \\ K'^{(2)} \end{bmatrix} = EK,$$
$$\begin{bmatrix} C_y^{(1)} & C_y^{(2)} \end{bmatrix} = C_y E^{-1}, \quad \begin{bmatrix} C_z^{(1)} & 0 \end{bmatrix} = C_z E^{-1}. \tag{21}$$

Note that the above transformation, which is known as a similarity transform[68], gives an equivalent model and thus equation (20) is still general and describes the same second order statistics for the observed time series y_k and z_k as equations (15) and (18) do.

Equation (20) can also be written in a form similar to equation (2) in **Methods**. To see this, note that by rearranging the first line of equation (20) to move $A'_{21} x_k^{(1)}$ to second term we get

$$\begin{bmatrix} x_{k+1}^{(1)} \\ x_{k+1}^{(2)} \end{bmatrix} = \begin{bmatrix} A'_{11} & 0 \\ 0 & A'_{22} \end{bmatrix} \begin{bmatrix} x_k^{(1)} \\ x_k^{(2)} \end{bmatrix} + \begin{bmatrix} K^{(1)} y_k \\ K'^{(2)} y_k + A'_{21} x_k^{(1)} \end{bmatrix}. \tag{22}$$

Here, $K'^{(2)}$ and A'_{21} can be thought of as one function applied to the concatenation of y_k and $x_k^{(1)}$. Equivalently, to get the computation graph depicted in **Supplementary Fig. 1a**, we can rewrite the formulation to have the lower block of the last term (i.e., $K'^{(2)} y_k + A'_{21} x_k^{(1)}$) as a function of y_k and $x_{k+1}^{(1)}$, the latter of which is the output of the first line of equation (22). To do so, we rearrange the terms as

$$\begin{aligned} K'^{(2)} y_k + A'_{21} x_k^{(1)} &= K'^{(2)} y_k + A'_{21} A'^{-1}_{11} A'_{11} x_k^{(1)} + A'_{21} A'^{-1}_{11} \left(K^{(1)} y_k - K^{(1)} y_k \right) \\ &= K'^{(2)} y_k + A'_{21} A'^{-1}_{11} \left(A'_{11} x_k^{(1)} + K^{(1)} y_k \right) - A'_{21} A'^{-1}_{11} K^{(1)} y_k \\ &= \left(K'^{(2)} - A'_{21} A'^{-1}_{11} K^{(1)} \right) y_k + A'_{21} A'^{-1}_{11} x_{k+1}^{(1)} \triangleq K^{(2)} \begin{bmatrix} y_k \\ x_{k+1}^{(1)} \end{bmatrix} \end{aligned} \tag{23}$$

where we assume that all behaviorally relevant states have associated dynamics in A'_{11} so that A'_{11} is nonsingular and we define $K^{(2)} \triangleq \left[\left(K'^{(2)} - A'_{21} A'^{-1}_{11} K^{(1)} \right) \quad A'_{21} A'^{-1}_{11} \right]$. Substituting equation (23) into equation (22) and writing it together with the second and third lines of equation (20) gives

$$\begin{cases} \begin{bmatrix} x_{k+1}^{(1)} \\ x_{k+1}^{(2)} \end{bmatrix} = \begin{bmatrix} A'_{11} x_k^{(1)} \\ A'_{22} x_k^{(2)} \end{bmatrix} + \begin{bmatrix} K^{(1)} y_k \\ K^{(2)} \begin{bmatrix} y_k \\ x_{k+1}^{(1)} \end{bmatrix} \end{bmatrix}. \\ y_k = C_y^{(1)} x_k^{(1)} + C_y^{(2)} x_k^{(2)} + e_k \\ z_k = C_z^{(1)} x_k^{(1)} + \epsilon_k \end{cases} \tag{24}$$

We can next add $C_z^{(2)} x_k^{(2)}$ to the third line of equation (24) for completeness to support the case of n_1 being smaller than the dimension required to cover all behaviorally relevant latent states (for example, the special case of $n_1 = 0$, which covers NDM as a special case). This provides an equivalent predictor form description of a general linear dynamical system model. Finally, to get the nonlinear two-section RNN model in DPAD, we can replace each parameter (e.g., $K^{(1)}$) in equation (24) with a general nonlinear function (e.g., $K^{(1)}(\cdot)$) to get equation (2) from **Methods**. These nonlinear functions/parameters can be implemented in the form of arbitrary multilayer neural networks (**Methods**). These nonlinear general parameters formulate a two-section RNN, which are learned with the 4-step DPAD learning approach (**Supplementary Fig. 1a**).

Supplementary Note 3: Distinction of multi-step optimization with a single optimization of a mixed objective

One way to encourage latent states to be behavior predictive within one optimization step is to form a mixed objective for neural and behavior data as

$$L = L_z + \lambda L_y = \sum_k NLL(z_k, \hat{z}_k) + \lambda \sum_k NLL(y_k, \hat{y}_k), \tag{25}$$

where $NLL(\cdot)$ denotes the negative log-likelihood of a prediction, and λ is a regularization parameter that determines how much attention is paid to each of the two terms in the objective, i.e., the behavior prediction loss denoted by L_z and the neural prediction loss denoted by L_y. If the goal was to maximize the total data likelihood for both neural and behavioral data without trying to dissociate the latent states and prioritize learning the behaviorally relevant ones, then single-step optimization of such a mixed objective with some non-zero weight λ—that needs to be selected based on the relative signal-to-noise ratio or reliability of the neural versus behavior data—would have been appropriate. But a key goal of DPAD is to instead achieve dissociation of behaviorally relevant versus other states, while prioritizing the learning of the former. To enable prioritized dissociation and also accurately learn all neural dynamics, whether behaviorally relevant or not, DPAD relies on both its architectural separation of latent states into $x_k^{(1)}$ and $x_k^{(2)}$ (as in equation (2) versus equation (1)) and its multi-step optimization procedure (**Supplementary Fig. 1**). If the latent states were not separated into two sections, there would be no "dissociation" since the same unified latent state vector would be capturing both behaviorally relevant and other neural dynamics. Conversely, even if the latent states were separated into two sections as in equation (2), but the learning only occurred in a single optimization step, it could be difficult to guarantee that one section of the latent state (i.e., $x_k^{(1)}$) would exclusively capture behaviorally relevant neural dynamics, leaving any remaining other dynamics to be captured by the other section (i.e., $x_k^{(2)}$).

In DPAD, to achieve our prioritized dissociation goal explained above, in optimization step 1, we learn $x_k^{(1)}$ by solely focusing on a behavior prediction objective to dissociate behaviorally relevant states and prioritize their learning. The objective of this optimization can be viewed as the extreme case of equation (25) where $\lambda = 0$ (**Supplementary Fig. 1a**). Then, in optimization step 3, we learn additional states $x_k^{(2)}$ by optimizing the prediction of any remaining neural dynamics as the objective. The objective of this optimization thus can be viewed as the extreme case of equation (25) with $\lambda \to \infty$ (**Supplementary Fig.**

1a). Thus, instead of using a regularization parameter like λ to impose a tradeoff between behavior and neural description within one set of latent states, DPAD allows the user to dedicate a subset of latent states ($x_k^{(1)}$) to solely focus on capturing behaviorally relevant neural dynamics ($\lambda = 0$) to achieve prioritization, while allowing the remaining latent states ($x_k^{(2)}$) to capture any remaining neural dynamics to achieve dissociation.

Our results across multiple datasets in this work (e.g., **Figs. 3-4, Supplementary Fig. 9**) suggest that the combination of DPAD's architectural separation of latent states (i.e., its two-section RNN model) and its multi-step optimization leads to models that better predict neural-behavioral data compared with existing alternative approaches. It would be interesting for future work to study whether and, if so, how enforcing dissociation of learned dynamics across the two sections of DPAD's model can also be done with a different learning approach, such as a single-step optimization.

Supplementary Note 4: extended caption for Extended Data Table 1

We provide an extended explanation for some columns of **Extended Data Table 1** below.

Input samples used to infer latent x_k: The subset of the input neural time series $\{y_1, y_2, \dots\}$ that are used to estimate the latent variable x_k associated with time sample k. Some methods (e.g., LFADS, TNDM, TAME-GP) aggregate information non-causally over a typically fixed-length finite window/segment of data of length T, often representing one trial: $x_k | y_1, y_2, \dots, y_T$. Recursive dynamic models (e.g., DPAD, NDM) causally aggregate information over all past neural samples: $x_k | y_1, y_2, \dots, y_{k-1}$. Static or semi-static methods extract x_k using one input sample $x_k | y_k$ (e.g., dPCA), or a fixed small window of input samples (e.g., $x_k | y_{k-5} \dots y_{k+4}$ for CEBRA).

Dynamic or static: Dynamic models have an explicit description of the temporal structure in data, which allows them to aggregate information over time. Recursive dynamic models including DPAD learn an explicit recursive description for the evolution of the latent dynamics. Sequential auto-encoders encode a fixed-length data window (e.g., a trial) into an initial condition, from which a dynamical system is initialized and generates the latent time series. Gaussian process models impose a model on temporal cross-correlation of latent states at different delays, without describing the evolution of latents recursively. In contrast to these, static models consider each given data sample on its own, and thus extract the same encoding regardless of the temporal order/structure of the input sequence. Convolutional models (e.g.,

CEBRA) consider each small data window on its own and can't aggregate information beyond that window, mostly similar to static models.

Prioritize behaviorally relevant neural dynamics: Methods that can incorporate the reconstruction of behavior from neural data as part of their learning objective, ideally with priority. Methods that consider both neural and behavioral data, typically do so with a mixed objective by adding behavior reconstruction to the overall objective as done in TNDM[18] for example. The mixed objective approach leads to a partial prioritization in the sense that the learned states are not guaranteed to be solely behaviorally relevant, i.e., focused on the behavior reconstruction (see **Supplementary Note 3**). DPAD in contrast ensures that behavior reconstruction is the sole objective when extracting the behaviorally relevant latent states, which are thus both dissociated from other neural states as well as prioritized in learning (that is, they are learned first).

Dissociate behaviorally relevant and other neural dynamics: DPAD is the only dynamic nonlinear method that learns both behaviorally relevant neural dynamics and other neural dynamics, and dissociates the two into separate latent states. As noted above, methods with a mixed objective (e.g., TNDM) do not guarantee that any subset of states are solely behaviorally relevant and do not contain other dynamics; thus, these methods do not dissociate the two types of states (**Supplementary Note 3**). Other behavior decoding methods (e.g., RNN decoders, LDA, SVM) only learn behaviorally relevant neural dynamics and do not learn any other neural dynamics, and thus cannot dissociate the two.

Learned reconstruction models: The reconstruction models that are natively learned by the method when extracting latents, in order to reconstruct neural or behavioral data from these learned latents. Some methods (e.g., CEBRA, TAME-GP, pi-VAE, dPCA) indirectly prioritize behaviorally relevant neural dynamics without learning a model to reconstruct behavior from the extracted latents. For all methods, a post-hoc regression model can be learned to map the extracted latents to neural/behavioral data, but here we list the reconstruction models that are natively learned by the method, i.e., learned when training the latents.

Supplementary References

111. LeCun, Y., Bengio, Y. & Hinton, G. Deep learning. *Nature* **521**, 436–444 (2015).
