# Supplementary material for: Dissociative and prioritized modeling of behaviorally relevant neural dynamics using recurrent neural networks
Source: Nat Neurosci. 2024 Sep 6;27(10):2033–45. doi: 10.1038/s41593-024-01731-2 (PMC11452342; doi:10.1038/s41593-024-01731-2)
Supplement: Supplementary file 2 — Reporting Summary [file 41593_2024_1731_MOESM2_ESM.pdf]

nature portfolio

Corresponding author(s): Maryam M. Shanechi

Last updated by author(s): 2024/07/10

Reporting Summary

Nature Portfolio wishes to improve the reproducibility of the work that we publish. This form provides structure for consistency and transparency in reporting. For further information on Nature Portfolio policies, see our Editorial Policies and the Editorial Policy Checklist.

Statistics

For all statistical analyses, confirm that the following items are present in the figure legend, table legend, main text, or Methods section.

n/a	Confirmed	
☐	☒	The exact sample size (*n*) for each experimental group/condition, given as a discrete number and unit of measurement
☐	☒	A statement on whether measurements were taken from distinct samples or whether the same sample was measured repeatedly
☐	☒	The statistical test(s) used AND whether they are one- or two-sided<br>*Only common tests should be described solely by name; describe more complex techniques in the Methods section.*
☐	☒	A description of all covariates tested
☐	☒	A description of any assumptions or corrections, such as tests of normality and adjustment for multiple comparisons
☐	☒	A full description of the statistical parameters including central tendency (e.g. means) or other basic estimates (e.g. regression coefficient) AND variation (e.g. standard deviation) or associated estimates of uncertainty (e.g. confidence intervals)
☐	☒	For null hypothesis testing, the test statistic (e.g. *F*, *t*, *r*) with confidence intervals, effect sizes, degrees of freedom and *P* value noted<br>*Give P values as exact values whenever suitable.*
☒	☐	For Bayesian analysis, information on the choice of priors and Markov chain Monte Carlo settings
☒	☐	For hierarchical and complex designs, identification of the appropriate level for tests and full reporting of outcomes
☒	☐	Estimates of effect sizes (e.g. Cohen's *d*, Pearson's *r*), indicating how they were calculated

Our web collection on statistics for biologists contains articles on many of the points above.

Software and code

Policy information about availability of computer code

Data collection	Dataset 1: Kinematic data were acquired using the Cortex software package (version 5.3) to track retroreflective markers in 3D (Motion Analysis, Inc USA). Joint angles were solved from the 3D marker data using a Rhesus macaque musculoskeletal model via the SIMM toolkit (version 4.0, MusculoGraphics Inc., USA).<br>Dataset 2: The visual stimuli in the task with saccadic eye movements were controlled via custom LabVIEW (version 9.0, National Instruments) software executed on a real-time embedded system (NI PXI-8184, National Instruments).<br><br>This data collection is also described in prior work:<br>- https://www.nature.com/articles/s41593-020-00733-0<br>- https://doi.org/10.1073/pnas.1504172112
Data analysis	Custom code (python version 3.9) for the DPAD algorithm is available online at https://github.com/ShanechiLab/DPAD

For manuscripts utilizing custom algorithms or software that are central to the research but not yet described in published literature, software must be made available to editors and reviewers. We strongly encourage code deposition in a community repository (e.g. GitHub). See the Nature Portfolio guidelines for submitting code & software for further information.

Data

Policy information about availability of data

All manuscripts must include a data availability statement. This statement should provide the following information, where applicable:

- Accession codes, unique identifiers, or web links for publicly available datasets
- A description of any restrictions on data availability
- For clinical datasets or third party data, please ensure that the statement adheres to our policy

Three of the datasets used in this work are publicly available (refs. 47–49,54). The other two datasets used to support the results are available upon reasonable request from the corresponding author. Source data are provided with this paper.

Research involving human participants, their data, or biological material

Policy information about studies with human participants or human data. See also policy information about sex, gender (identity/presentation), and sexual orientation and race, ethnicity and racism.

Reporting on sex and gender	This study did not involve human participants.
Reporting on race, ethnicity, or other socially relevant groupings	This study did not involve human participants.
Population characteristics	This study did not involve human participants.
Recruitment	This study did not involve human participants.
Ethics oversight	This study did not involve human participants.

Note that full information on the approval of the study protocol must also be provided in the manuscript.

Field-specific reporting

Please select the one below that is the best fit for your research. If you are not sure, read the appropriate sections before making your selection.

☒ Life sciences ☐ Behavioural & social sciences ☐ Ecological, evolutionary & environmental sciences

For a reference copy of the document with all sections, see nature.com/documents/nr-reporting-summary-flat.pdf

Life sciences study design

All studies must disclose on these points even when the disclosure is negative.

Sample size	A sample size of five non-human primate subjects was used, consisting of one non-human primate subject for each of the five behavioral tasks. This is comparable with the sample sizes reported in previous non-human primate neurophysiology publications (e.g., https://www.nature.com/articles/s41593-020-00733-0). All results held for all subjects.
Data exclusions	No data was excluded from the study.
Replication	Results were replicated in all subjects performing all experimental tasks and all attempts at replication were successful.
Randomization	Not relevant for this study. Identical analyses were performed on data from each subject and the results were reported for each subject. There was no grouping of subjects.
Blinding	Not relevant for this study. There was no group allocation.

Reporting for specific materials, systems and methods

We require information from authors about some types of materials, experimental systems and methods used in many studies. Here, indicate whether each material, system or method listed is relevant to your study. If you are not sure if a list item applies to your research, read the appropriate section before selecting a response.

Materials & experimental systems

n/a	Involved in the study
☒	☐ Antibodies
☒	☐ Eukaryotic cell lines
☒	☐ Palaeontology and archaeology
☐	☒ Animals and other organisms
☒	☐ Clinical data
☒	☐ Dual use research of concern
☒	☐ Plants

Methods

n/a	Involved in the study
☒	☐ ChIP-seq
☒	☐ Flow cytometry
☒	☐ MRI-based neuroimaging

Animals and other research organisms

Policy information about studies involving animals; ARRIVE guidelines recommended for reporting animal research, and Sex and Gender in Research

Laboratory animals	Main datasets (datasets 1-4): four adult male rhesus macaques (macaca mulatta) ages 5 (subject J), 8 (subject A), 10 (subject T), and 11 (subject I) years old.
Wild animals	This study did not involve wild animals.
Reporting on sex	Main datasets (datasets 1-4) were from four adult male rhesus macaques.
Field-collected samples	This study did not involve field-collected samples.
Ethics oversight	For each dataset, all animal procedures were performed in compliance with the National Research Council Guide for Care and Use of Laboratory Animals and were approved by the Institutional Animal Care and Use Committee at the respective institution, namely New York University (datasets 1 and 2), Northwestern University (datasets 3 and 5), or University of California San Francisco (dataset 4).

Note that full information on the approval of the study protocol must also be provided in the manuscript.
